# Validation of experimental charge-density refinement strategies: when do we overfit?

**DOI:** 10.1107/S2052252517005103

**Published:** 2017-05-24

**Authors:** Lennard Krause, Benedikt Niepötter, Christian J. Schürmann, Dietmar Stalke, Regine Herbst-Irmer

**Affiliations:** a Institut für Anorganische Chemie, Universität Göttingen, Tammannstraße 4, Göttingen 37077, Germany

**Keywords:** charge density, cross-validation, error detection, *R*_free_, refinement strategies

## Abstract

A cross-validation method is supplied to judge between various strategies in multipole refinement procedures. Its application enables straightforward detection of whether the refinement of additional parameters leads to an improvement in the model or an overfitting of the given data. For all tested data sets it was possible to prove that the multipole parameters of atoms in comparable chemical environments should be constrained to be identical. In an automated approach, this method additionally delivers parameter distributions of *k* different refinements. These distributions can be used for further error diagnostics, *e.g.* to detect erroneously defined parameters or incorrectly determined reflections. Visualization tools show the variation in the parameters. These different refinements also provide rough estimates for the standard deviation of topological parameters.

## Abstract 2

A cross-validation method is supplied to judge between various strategies in multipole refinement procedures. It easily detects when the refinement of additional parameters leads to an improvement in the model or when it simply overfits the given data.

## 1. Introduction   

Although Philip Coppens (2005 ▸) wrote that ‘Charge densities [have] come of age’, experimental charge-density studies still depend heavily on the amount and quality of the measured data. Additionally, there is no published investigation of whether or not the refinement of all possible parameters might lead to overfitting of the data, because the old benchmark of the ‘independent atom model’ (IAM), which should not run below the 10:1 data-to-parameter ratio to keep the problem sufficiently over-determined, is hardly violated even with a demanding charge-density investigation. In a routine IAM refinement only nine parameters, three positional and six anisotropic displacement parameters, are necessary to model the structure, while in a multipole refinement (MM) *via* the Hansen–Coppens formalism (Hansen & Coppens, 1978 ▸) many more parameters are needed to describe the asphericity of the electron density distribution
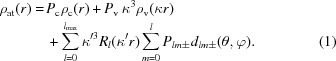
Here, the density is divided into a core density, a spherical valence density and an aspherical valence density. The parameters κ and κ′ are used to describe the expansion and contraction of the density. This approach requires up to 27 (= 25 + 2) additional parameters per atom for multipole populations up to the hexadecapole level, and the κ and κ′ parameters. Some of the parameters are highly correlated, like the monopole population and the κ parameter of one particular atom. The valence density is mainly described by low-order data, but to derive proper thermal displacement parameters high-resolution low-temperature data are needed. Due to the development of more intense X-ray sources, more sensitive area detectors and improved cryogenic crystal cooling techniques, the data quality has improved significantly so that even an anharmonic description of the thermal motion is increasingly reported in the literature (see *e.g.* Dos Santos *et al.*, 2016 ▸; Poulain *et al.*, 2014 ▸; Domagała *et al.*, 2014 ▸), requiring even more parameters per atom. In experimental charge-density investigations, high-resolution data up to at least 1.0 Å^−1^ in sin(θ)/λ are necessary. Therefore, the data-to-parameter ratio is usually higher than for a routine IAM refinement. This ratio alone might suggest that overfitting is not of any concern in experimental charge-density determinations. However, this is not necessarily true because not all reflections contribute to all parameters equally, *e.g.* only the low-order reflections determine the valence density. Consequently, the increasing number of parameters might result in a significant drop in the *R* value without improving the model. A statistical method to detect that phenomenon is cross-validation. In this model-validation technique, a sample population of data is divided into complementary subsets. One subset (the ‘training’ or ‘work’ set) is used to derive a model, while the other is used to validate the model (the ‘validation’ or ‘test’ set). In macromolecular crystallography this is known as the *R*_free_ concept (Brünger, 1992 ▸, 1997 ▸). Here, the measured data are divided into a work set (often 95–90% of all data) and a test set (the remaining 5–10%). The model is refined against the work set, while the test set is never used for refinement but only to calculate an *R* value, the *R*_free_ value. Adding parameters will lead to a decrease in *R*_work_, but only sensible added parameters will also decrease *R*_free_. An increasing *R*_free_ is an unerring sign of overfitting. After determination of all sensible parameters, a final refinement can then be performed against all data.

In charge-density investigations, the following points need to be addressed:

(i) To adjudicate on overfitting, just the differences in *R*_free_ and *R*_work_ after introducing more parameters are monitored. Hence, we are not interested in their absolute values, which is different to the approach of protein crystallographers.

(ii) Determination of *R*_work_ and *R*_free_ in different resolution shells can be helpful because different regions of reciprocal space are of varying importance for different kinds of parameter.

(iii) The number of reflections in the validation set can be crucial. If the number is too small, the standard deviation of *R*_free_ is too large. The standard deviation of the free *R* value is given approximately by *R*_free_/(2*n*)^1/2^ (Tickle *et al.*, 2000 ▸), where *n* is the number of reflections in the test set. As the *R* values, and especially the differences in the *R* values, are very small, this standard deviation is normally too high to provide a conclusive answer from a single *R*_free_ value. If *n* is too large, the completeness of the training set is too small, leading to biased models. In statistics, this is solved by *k*-fold cross-validation, which means that the sample is divided into *k* subsets. One subset is used as a test set, while the other *k* − 1 sets give the model. This process is repeated *k* times with a different subset as the test set. Hence, all data are used for both model deriving and validation. For a charge-density analysis this means that *e.g.* 20 test sets are produced. Every reflection is considered once in a test set and otherwise in the work set. This method was previously employed to validate the weighting of restraints (Paul *et al.*, 2011 ▸; Zarychta *et al.*, 2011 ▸) but should also be valid to judge *e.g.* local symmetry constraints, as the program *XD* (Volkov *et al.*, 2006 ▸) does not offer the opportunity to introduce restraints. A final refinement is then of course employed against all available data. Out of the *k* different refinements the mean values 〈Δ*R*_free_〉 can be calculated. Instead of checking 〈Δ*R*_free_〉, a new value *R*_cross_ is defined that takes into account all test sets of all *k* refinements. Here, all differences between *F*_o_ and *F*_c_ for all reflections as validation reflections are used, therefore it is the *R*_free_ for *all* reflections as validation
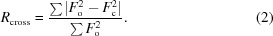
A similar procedure has recently been described for macromolecules (Luebben & Gruene, 2015 ▸).

(iv) Normally the test set is chosen randomly, but caution is to be advised in non-centrosymmetric space groups or if pseudo-symmetry is present, as the Friedel pairs cannot be considered to be independent observations. The Friedel pairs or pseudo-symmetrically related reflections must be either both in the training set or both in the validation set.

(v) The validation set must be unbiased. That means it must never be used for the refinement unless the parameters are ‘shaken’ before the cross-validation refinement starts.

For the *k* different models, the distribution of each refined parameter *v* can be checked and compared with the value *v*_total_ including its s.u., *s*_total_, derived from a refinement against the complete data set. Model bias by omission of data can then be easily identified by outliers, *e.g.* values *v*_*i*_ that deviate by more than three times *s*_total_ from *v*_total_. If all *k* refinements are independent, one should find a normal distribution with a mean value *v*_mean_ that is identical to *v*_total_, and a standard deviation *s*_mean_ that equals *s*_total_ divided by a correction factor that can be derived from Cochran’s theorem (Cochran & Wishart, 1934 ▸), *e.g.* 0.973 for ten test sets, 0.987 for 20 test sets and 0.995 for 50 test sets. However, as each refinement uses (*k* − 1)/*k* × *n*_d_ reflections of the total *n*_d_ data, the refinements are not independent and therefore the expected standard deviation *s*_mean_ is smaller than *s*_total_. As a converse argument, *s*_mean_ > *s*_total_ indicates problems in the refinement.

Subsequent to a refinement with no clear indication of overfitting, the distribution of topological QTAIM (quantum theory of atoms in molecules; Bader, 1990 ▸) parameters like the density ρ, Laplacian ∇^2^ρ and ellipticity ∊ at the bond critical points should also be checked. Following the same arguments as above, we can estimate lower limits for the standard uncertainties of these sensitive values that are otherwise either entirely unavailable or include severe limitations (see the software manual for *XD*).

## 2. Method   

A Python script was developed that runs most of the required steps automatically. As input, it needs an IAM model including hydrogen-atom positions, the *XD* master files (complete strategy), a parameter file and the data file. A stepwise addition of parameters, following the suggestions made in the *XD* manual, is highly recommended. Apart from convergence issues, a smaller step (*e.g.* smaller groups of parameters) supports the successful recognition of an overparameterization, along with the possibility of actually finding parameters that are likely to contribute to the overfitting of the data (see supporting information).

In this automatic process, the merged data set is first divided randomly into *k* (normally 20) different training and validation sets.

(i) For each training set as well as for the total data set the following steps are performed.

(*a*) In *SHELXL* (Sheldrick, 2008 ▸, 2015 ▸):

(1) Random shifts are applied to all coordinates and *U_ij_* values;

(2) A high-order refinement of the heavy-atom positional and anisotropic displacement parameters is performed to reduce bias;

(3) A low-order refinement with fixed heavy atoms follows;

(4) The residual density peaks are automatically assigned to hydrogen atom positions.

(*b*) In *XD* (Volkov *et al.*, 2006 ▸) the full MM refinement strategy is performed. In the first step the H-atom distances are adjusted to the neutron diffraction values (Allen & Bruno, 2010 ▸).

(*c*) For every refinement step, a zero-cycle structure factor calculation against the free sets (*R*_free_) is performed.

(ii) *R*_cross_ is calculated by combining all xd.fco files of all validation sets for all steps.

(iii) Differences in 〈*R*_work_〉 and *R*_cross_ are calculated for the individual steps and represented graphically.

(iv) In a second process, the distributions of all refined parameters of the *k* refinements with mean values *v*_mean_ and standard deviation *s*_mean_ are compared with the values of the refinement against all data, *v*_total_, and the estimated standard uncertainty, *s*_total_, calculated by *XD*. Individual refinement steps are selected for this comparison.

(*a*) To check for normal distribution, the Shapiro–Wilk test (Shapiro & Wilk, 1965 ▸) is performed for every parameter and the *W* and *p* values are given. (In this test two values, *W* and *p*, are calculated. *W* can be interpreted as a correlation coefficient, so has a value between 0 and 1. If the value is larger than a defined *W*_crit_, the hypothesis of a normal distribution is accepted. *p* describes the probability of this particular sample distribution under the assumption of a normal distribution.)

(*b*) A complete list of all refined parameters is given, with *v*_mean_, *s*_mean_, *v*_total_, *s*_total_, *W* and *p*.

(*c*) Several tables for the diagnostics of outliers are given:

(1) A list of all parameters *v*_*i*_ with |*v*_total_ − *v*_*i*_| > 3*s*_total_;

(2) A list of all parameters from the Shapiro–Wilk tests with a *W* value smaller than 0.905 or a *p* value smaller than 0.05, which means that the hypothesis of a normal distribution is refused with a significance level of 0.05;

(3) A list of all parameters with |*v*_total_ − *v*_mean_|/*s*_total_ > 0.5;

(4) A list of all parameters with *s*_mean_ > *s*_total_;

(5) A summary of the number of outlier parameters per test set.

(*d*) For every parameter, a plot can be produced showing the refinement against all data in grey and the distribution of the *k* refinements in red (see Fig. 4).

(*e*) To visualize the variation in electron density of the *k* refinements, an error cube is mapped on a density cube. A colour-coded overlay (*e.g.* transparent to red) of such an error cube on a density cube calculated from the full model will highlight regions of higher uncertainties (see Fig. 6). [A density cube is calculated for all *k* refinements and for the complete model using the *XDPROP* module. The standard deviation of every grid point is calculated considering all *k* density cubes. A new cube containing only the deviation of each respective grid point is written. This cube is plotted as a colour-coded overlay (*e.g.* as a gradient from transparent to red) on specified iso-surface levels (1.0, 1.5, 2.0, 2.5) of the cube derived from the complete set of data. H atoms are automatically excluded from the calculation of the density cube because of their influence on the density combined with their unreliably determined positions that would add only a little information and clearly distract from the important parts.]

(v) Additionally, a routine can be started that runs *XDPROP* to search for bond critical points for all test sets at individually selectable steps of the whole refinement strategy.

(*a*) Again, the distribution of the *k* refinements is compared with the refinement against all data. From this distribution lower limits for standard deviations can be estimated.

(*b*) The Shapiro–Wilk values *W* and *p* are calculated.

This procedure will now be explicitly described for benchmark structure **1**. Subsequently, benchmark structure **2** will be discussed much more briefly. Finally, some features of three additional structures will be presented to illustrate how this method can help to detect errors in the refinement strategy or in the data. These features emphasize, among others, the need for chemical constraints to limit pole populations of chemically equivalent atoms. The structures themselves and their topological analyses are not within the scope of the current paper and will be discussed elsewhere.

## 3. Experimental   

### 3.1. Benchmark structure **1**   

**Figure 1:**
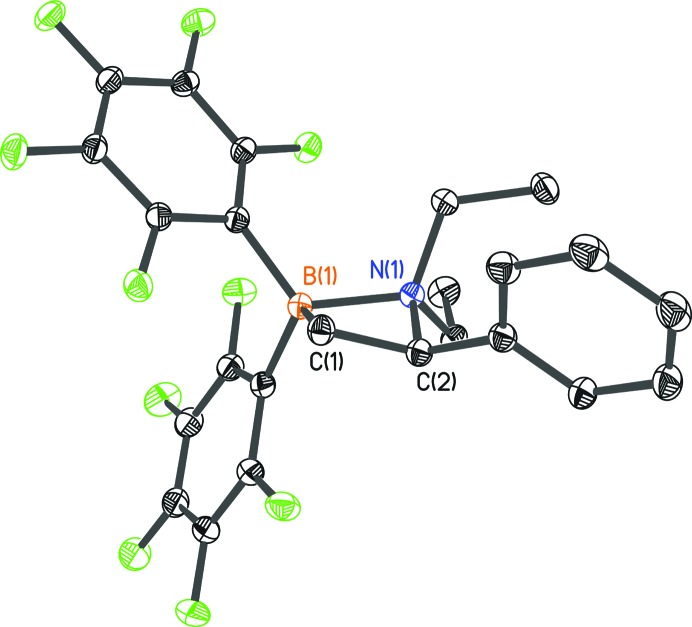
The molecular structure of **1**. Anisotropic displacement parameters are depicted at the 50% probability level. H atoms have been omitted for clarity.

Data for **1** (Schwendemann *et al.*, 2011 ▸; Fig. 1 ▸) were collected on a Bruker D8 three-circle goniometer equipped with a Bruker TXS-30 Mo rotating anode with INCOATEC Helios mirror optics and an APEXII detector at 100 K. The compound crystallizes in space group 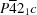 with one molecule in the asymmetric unit.

The local coordinate systems were defined in such a way that the highest possible symmetry could be applied. This led to cylindrical symmetry for the H and F atoms and mirror symmetry for the ethyl and phenyl C atoms. For the *para*-C atoms even *mm*2 symmetry was adopted (for details see the supporting information). We constrained the pole population of chemically equivalent atoms to be identical (chemical constraints; for details see the supporting information). The two main questions we posed were:

(i) Can the local symmetry constraints be released without overfitting and would this add information to the model?

(ii) Can the chemical constraints be released without overfitting and would this cause differences in the parameters concerning the constrained atoms?

#### 3.1.1. Calculation of *R*_cross_   

The Friedel pairs were not merged because the structure crystallizes in a noncentro­symmetric space group. For all validation and training sets, it was ensured that both Friedel mates were in the same set.

**Figure 2:**
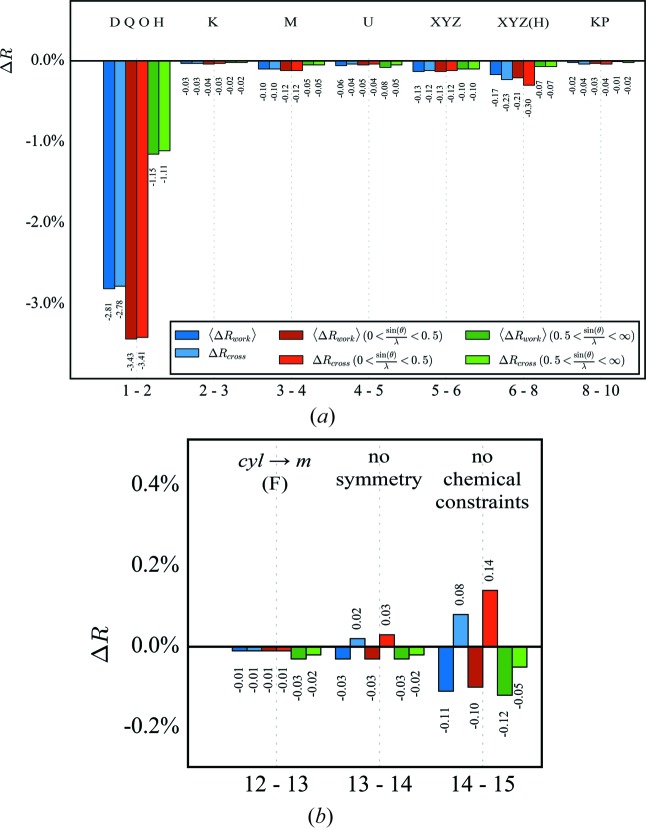
Δ*R* values for the initial refinement strategy. Abbreviations: M indicates monopoles, D dipoles, Q quadrupoles, O octupoles, H hexadecapoles, U *U_ij_*, K κ and KP κ′. A detailed description of the strategy is given in the supporting information.

〈Δ*R*_work_〉 and Δ*R*_cross_ were calculated for all data and for reflections on either side of sin(θ)/λ = 0.5 Å^−1^ to get a feeling for the influence of low- and high-order reflections. Fig. 2 ▸ shows the results.

The first step in *XD* is refining multipole populations. Both 〈*R*_work_〉 and *R*_cross_ drop significantly. For both residual values, the improvement is, as expected, larger from the low-order reflections compared with the high-order ones, because the valence density is mainly described by low-order reflections. The refinement of the κ parameters has only a marginal effect on the *R* values but seems to improve the model slightly. Adding the monopole populations shows the expected effect: an improvement mainly from the low-order reflections. In addition, the following adjustments of displacement and positional parameters again do not indicate any overfitting. Here, a clear difference between low- and high-order reflections is not expected. The adjustment of the H-atom positional parameters in a low-order refinement followed by refinement of all other parameters against all data is a major improvement and is, as expected, mainly due to the low-order reflections. The refinement of κ′ shows only a marginal effect on the *R* values.

**Figure 3:**
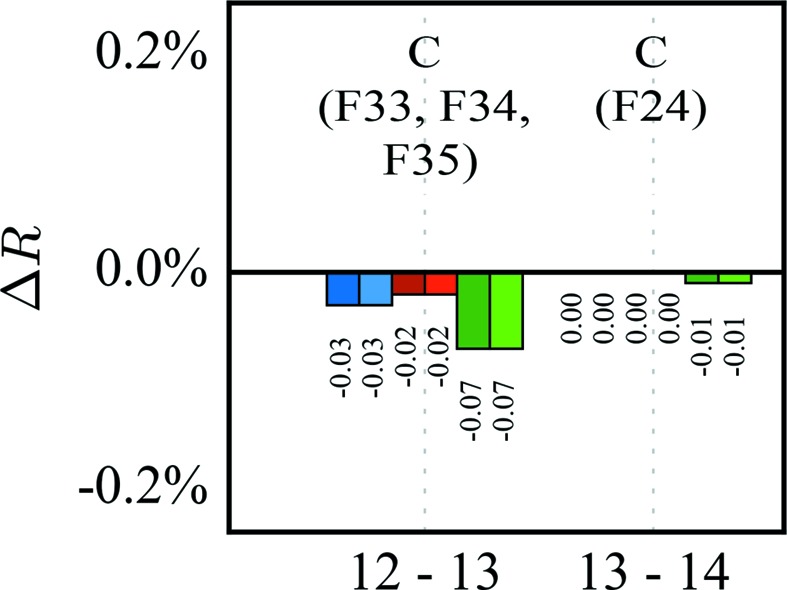
*ΔR* values checking the anharmonic refinement of several F atoms. C denotes third-order Gram–Charlier coefficients.

The next two steps concern the local symmetry constraints. First, the local symmetry of the F atoms is lowered from cylindrical to mirror plane symmetry. The small effect on the *R* values indicates neither a real model improvement nor clear overfitting. Although the changes are subtle, it seems that the high-order reflections unexpectedly contribute the most. Refining without any symmetry constraints, however, increases *R*_cross_ slightly, indicating overfitting of the data, although the total data-to-parameter ratio of 27.0 still seems to be at the safe side, while the ratio of low-order data to the sum of monopole, multipole and κ parameter is only 5.1. Overfitting is even more evident when the chemical constraints for equivalent atoms are also abandoned. Even here, the data-to-parameter ratio is still 16.7, while the ratio of low-order data to the sum of monopole, multipole and κ parameter is now only 2.5. Anyway, the residual density maps seem to be improved, because some peaks close to F atoms vanish. Although releasing constraints on the pole populations is overfitting, nevertheless the residual density still needs improvement. Hence, an anharmonic description of the thermal motion of these atoms was tested as an alternative, because we observe the typical positive and negative shashlik-like density distribution (Herbst-Irmer *et al.*, 2013 ▸). Instead of releasing constraints, a refinement with third-order Gram–Charlier coefficients for four F atoms was performed in two steps (see Fig. 3 ▸). First, the three fluorine atoms F33, F34 and F35 of one C_6_F_5_ substituent and, in a second step, the *para*-F atom F24 of the second phenyl ring were anharmonically refined. Neither step shows any sign of overfitting and, as expected, the high-order reflections contribute the most. In the paper by Herbst-Irmer *et al.* (2013 ▸) we have already investigated preliminary *R*_free_ tests to validate the refinement of Gram–Charlier co­efficients but came to the conclusion that the results were not clear. Now, the *k*-fold cross-validation and the differences in *R*_cross_ instead of 〈*R*_free_〉 are much more decisive.

It is important to note that several criteria must be fulfilled for a physically reasonable refinement of anharmonic motion (Herbst-Irmer *et al.*, 2013 ▸):

(i) The residual density after harmonic refinement shows a positive and negative shashlik-like density distribution close to the atomic positions that vanishes after anharmonic refinement (see the supporting information).

(ii) For each anharmonically refined atom, at least one Gram–Charlier coefficient is larger than three times its s.u. (see the supporting information).

(iii) Kuhs’ rule (Kuhs, 1992 ▸) should be fulfilled (see the supporting information), at least for light elements like carbon and fluorine. [Kuhs introduced a rule for estimating the minimum data resolution for meaningful refinement of anharmonic thermal parameters (Gram–Charlier coefficients) for each anisotropic atom: *Q_n_* = 2*n* ^1/2^(2π)^−1/2^(2ln2)^1/2^〈*u* ^2^〉^−1/2^.]

(iv) The probability density function (pdf) should be reasonable. Unfortunately, the graphical representations presented by Herbst-Irmer *et al.* (2013 ▸) were obtained with a version of the program *MoleCoolQt* (Hübschle & Dittrich, 2011 ▸) that had a bug, producing wrong representations of the pdfs. Except for very strong anharmonic behaviour, normally no deviation from the harmonic ellipsoids is visible. However, the amount of negative density should be checked. For the two atoms F33 and F35 it is very small (lowest pdf values −0.87 and −0.34, respectively, and total integrated negative probability 0.000%), while for atoms F34 and F24 the lowest pdf values are −52.24 and −105.38, respectively, and the total integrated negative probabilities are 0.026 and 0.046%, respectively. These two atoms are chemically equivalent and therefore their monopole and multipole populations were constrained to be the same. Additionally, there is a high correlation between the Gram–Charlier coefficient *C*_222_ of atom F24 and the bond-directed dipole population of 90%, indicating that this anharmonic refinement is not reasonable. Therefore only atom F34 should be refined anharmonically, while atom F24 stays harmonic. This improves the pdf for F34 considerably (lowest pdf value −1.06 and total integrated negative probability 0.000%). Now the highest correlation of 60–70% for the Gram–Charlier coefficent is to the positional parameters *x*, *y* and *z*, as described previously (Herbst-Irmer *et al.*, 2013 ▸).

This example emphasizes that a small drop in *R*_cross_ is a necessary, but by no means a sufficient, condition for a physically reasonable model. After the anharmonic refinement of these three F atoms, we checked again the effect of local symmetry lowering (see the supporting information). A reduction of cylindrical to mirror plane symmetry for the F atoms still does not indicate overfitting and has no significant effect on 〈*R*_work_〉, but there is a slightly greater effect from the high-order reflection. As the influence on the residual density is also marginal [a drop in *e*_gross_ (Meindl & Henn, 2008 ▸) from 39 to 38.8 e Å^−3^], we decided that the final refinement strategy should maintain the above-mentioned local symmetry, should keep all possible chemical constraints and should contain the anharmonic refinement of the three F atoms.

#### 3.1.2. Distribution of the refined parameters   

**Figure 4:**
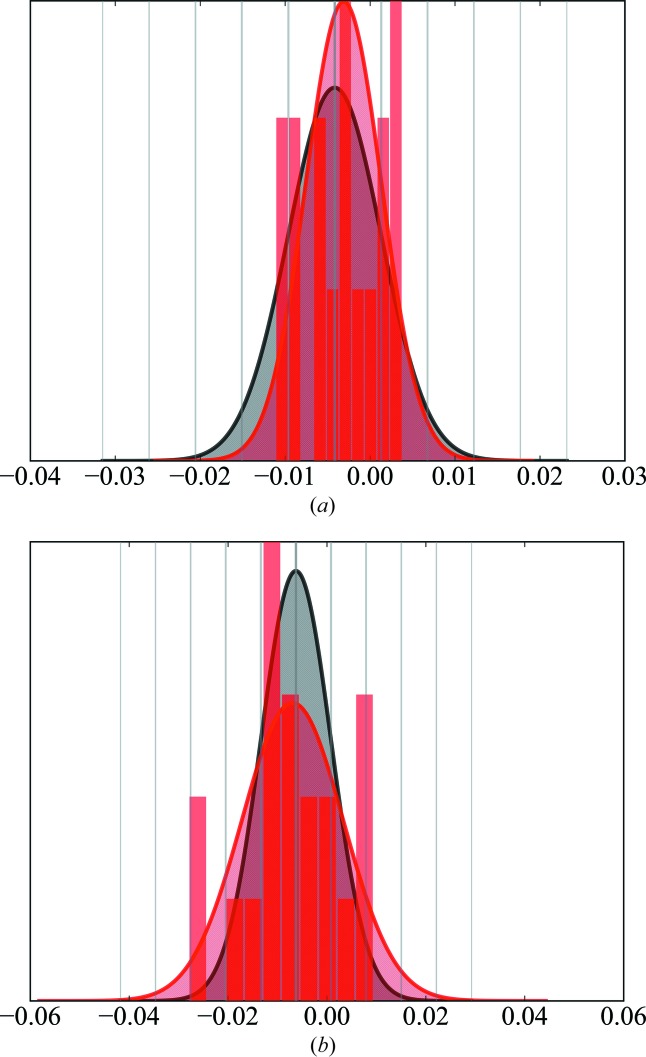
Distributions of two example parameters *v*, *e.g.* *P*_20_ and *P*_43+_ of atom C1. (*a*) A parameter with all values within *v*_total_ ± *s*_total_. (*b*) A parameter with one outlier. The distributions are plotted both as histogram bars and as fitted Gaussian functions. The value derived from the refinement against all data *v*_total_ with the estimated standard uncertainty *s*_total_ is depicted in grey, while the distribution of the value with *v*_mean_ and standard deviation *s*_mean_ of the refinements against the 20 different training sets is given in red.

To prevent model bias due to omission of reflections, we checked the distribution of parameters derived by the 20 different refinements. Fig. 4 ▸ shows two examples, in part (*a*) a parameter with all values within *v*_total_ ± *s*_total_ and in part (*b*) a parameter with one outlier.

For only nine out of 618 parameters of the final refinement did we find one to three such outliers. The entire set of 14 deviations do not belong to one particular training set (for details see the supporting information). Therefore, model bias due to omission of data can be excluded. Nine of the entire set of 14 outliers are octupole or hexadecapole populations of atoms C1 and C2. These atoms are refined without any local symmetry or chemical constraints. Therefore, we increased the adopted local symmetry in a new refinement strategy: we now adopt *m* symmetry for atoms C1, N1 and B1, and *mm*2 symmetry for all phenyl C atoms. Additionally, we started refining the multipole population only up to the octupole level and added hexadecapole populations in a later step.

**Figure 5:**
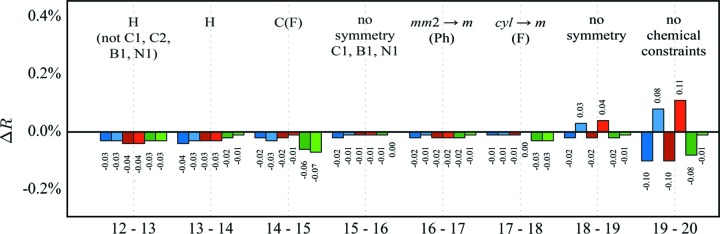
*ΔR* values for an improved refinement strategy. Abbreviations: H indicates hexadecapoles and C third-order Gram–Charlier coefficients.

Fig. 5 ▸ shows the Δ*R* values for this stepwise refinement. The hexadecapole populations are first refined for all atoms besides C1, C2, B1 and N1, and then for all atoms. Then the three F atoms are refined anharmonically. In the next two steps no symmetry constraints are applied to atoms C1, B1 and N1, and afterwards the phenyl atoms are relaxed from *mm*2 to *m* symmetry. None of these steps seems to overfit. In the next step, the local symmetry of the F atoms is changed from cylindrical to mirror plane symmetry. Again, no clear overfit is visible, but as before the high-order reflections seem to contribute the most. Releasing the local symmetry and chemical constraints in the last two steps clearly overfits. Therefore, we decided to stop the strategy after step 17 with the symmetry reduction of the phenyl atoms to *m* symmetry. For this last non-overfitting step there are only two parameters with |*v*_total_ − *v*_*i*_| > 3*s*_total_ with a maximum value of 3.3*s*_total_, and only seven parameters with *s*_total_ < *s*_mean_. The largest (*s*_mean_ − *s*_total_)/*s*_total_ is 0.34. From the parameters describing the valence density (monopole and multipole populations and κ values), the hypothesis of a normal distribution is confirmed with a significance of 0.05 for 235 of the 255 parameters.

**Figure 6:**
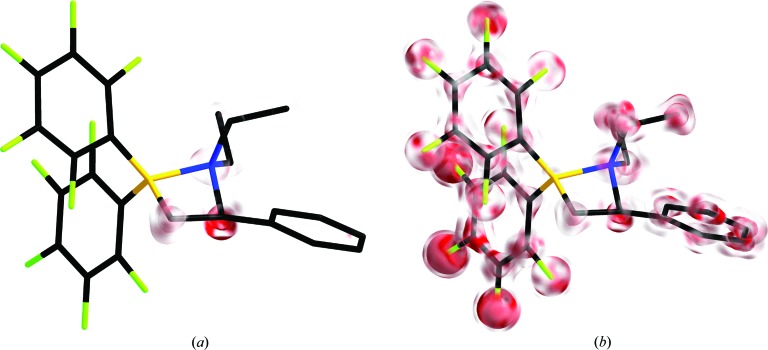
The variation in electron density for the *k* refinements, displayed as an error cube mapped on a density cube calculated for the complete model. Cubes are shown for (*a*) the last reasonable step 17 and (*b*) the refinement without any constraints (step 20). Two isosurface levels are plotted, at 1 and 2 e Å^−3^, and the standard deviation is capped at 0.015 e Å^−3^. Hydrogen density has been omitted.

The idea presented in the following is based on the assumption that overfitted parameters are imprecisely determined and therefore lead to higher variations in the electron density described by them. A density cube at a relevant isosurface level will not be able to show that variation, as the model parameters do not directly reflect this uncertainty. A computational detour involving a set of density cubes calculated for all work sets allows calculation of the standard deviation of each density grid point (see §2). A colour-coded overlay (*e.g.* transparent to red) of such an error cube on a density cube (calculated from the full model) is able to highlight regions of higher uncertainties (Fig. 6 ▸). This error cube is related to the σ(ρ) cube, the calculation of which is implemented in *XD*, although it shows additional features not covered hitherto. As the H-atom positions are by far the least precisely determined parameters, their density will probably show severe features in the corresponding error cube.

Fig. 6 ▸ compares the variation in the electron density of the *k* refinements in an error cube mapped on a density cube (see §2) for the last reasonable step 17 (Fig. 6 ▸ *a*), with step 20 (Fig. 6 ▸ *b*) refining all possible multipoles. For step 17, the atoms with the highest variations are those that are refined with no local symmetry constraints and no chemical constraints. Astonishingly, the refinement of atom B1 seems to be very stable. In step 20 all atoms show relatively high variation.

#### 3.1.3. Distribution of properties at bond critical points   

With this refinement strategy, the distribution of properties at bond critical points can now be evaluated. The calculation of errors on ρ and ∇^2^ρ in *XD* has some severe limitations, as mentioned in the manual. For the ellipticity ∊ no errors are provided. Nevertheless, an estimation of these errors would be useful. The distribution of *k* refinements can be used and, as previously described, the standard deviation derived from the distribution can be considered as a lower limit. The true error could be higher, because the *k* refinements are not independent. Table 1 ▸ shows a list of the properties at the bond critical points of the B—C and B—N bonds, comparing the final refinement with the first refinement without any local symmetry or chemical constraints.

The following conclusions can be drawn:

(i) For all properties, the differences between the value derived from the refinement against all data and the mean value of the 20 refinements is insignificant, |*v*_mean_ − *v*_total_|/*s*_total_ < 0.6.

(ii) For the density ρ, the estimated standard uncertainty for the refinement against all data is slightly larger than the standard deviation of the distribution of the 20 refinements, *s*_mean_/*s*_total_ < 1. The estimated standard uncertainty is in the range 0.009–0.012 e Å^−3^, or 1–1.5% of ρ. In an investigation using 13 different data sets of oxalic acid (Kamiński *et al.*, 2014 ▸), the distribution has a standard deviation in the range 0.03–0.06 e Å^−3^ or 1.5–3.0%.

(iii) For the Laplacian ∇^2^ρ, the estimated standard uncertainty is much smaller than the standard deviation of the distribution, *s*_mean_/*s*_total_ > 7.1. The standard deviations are between 0.3 and 0.6 e Å^−5^ *i.e.* 5–32% of *v*_total_. In the above-mentioned investigation (Kamiński *et al.*, 2014 ▸), the standard deviation is between 1 and 7 e Å^−5^, *i.e.* 6–28%.

(iv) For the ellipticity ∊, the standard deviation is between 0.009 and 0.02. In the work by Kamiński *et al.* (2014 ▸) it is between 0.01 and 0.04.

(v) Comparing the two refinements, most properties are very similar, but the Laplacian of the N—B bond changes from −0.94 to 1.86 e Å^−5^ and the ellipticity ∊ changes from 0.5 to 0.7. To investigate which refinement step is mainly responsible for these differences, all properties were checked for several steps. The most important point seems to be the use of chemical constraints, agreeing with the greatest effect on *R*_cross_ (for details see the supporting information).

#### 3.1.4. Influence of the number of test sets on the model   

The influence of the number of training sets on the models was also checked. We repeated the refinement using ten or 50 training sets, respectively (10% and 2% of data left out, respectively). The behaviour of *R*_cross_ is nearly identical for ten, 20 or 50 training sets (see the supporting information) but, as expected, for ten validation sets (more data left out) there are a higher number of outliers with |*v*_*i*_ − *v*_total_| < 3*s*_total_, while there are none for 50 validation sets. Accordingly, the standard deviations of the distributions shrink from ten to 50 validation sets. Here, we decided to use 20 sets, as this seems to be a sensible compromise between model bias and reasonable standard deviations.

#### 3.1.5. Influence of Friedel mates on the model   

**Figure 7:**
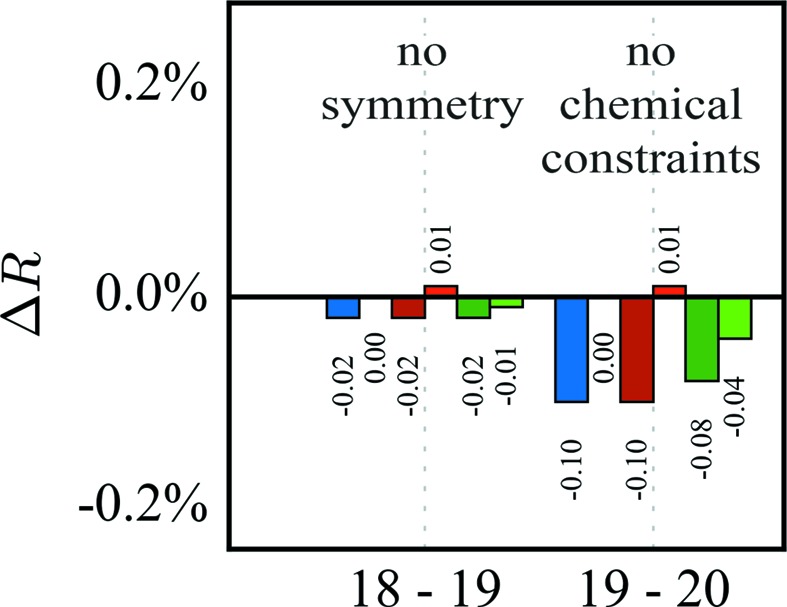
*ΔR* values for training sets with no special treatment of Friedel pairs

We ensured that Friedel pairs are either both in the training set or both in the validation set because structure **1** crystallizes in a non-centrosymmetric space group. To evaluate the impact of this treatment we performed the same refinement, but this time with randomly prepared training and validation sets without any special care for the Friedel pairs (see Fig. 7 ▸). Now overfitting is not that easy to detect. If we now check the feasibility of releasing the local symmetry or chemical constraints, 〈*R*_work_〉 responds in the familiar way but *R*_cross_ remains nearly unchanged. There is still an indication of overfitting if *R*_mean_ decreases much more than *R*_cross_, but the picture is much less obscure with a proper treatment of the Friedel pairs. As they are not independent, neither is the model independent of a particular Friedel mate being present or not in the training set.

### 3.2. Benchmark structure 2   

**Figure 8:**
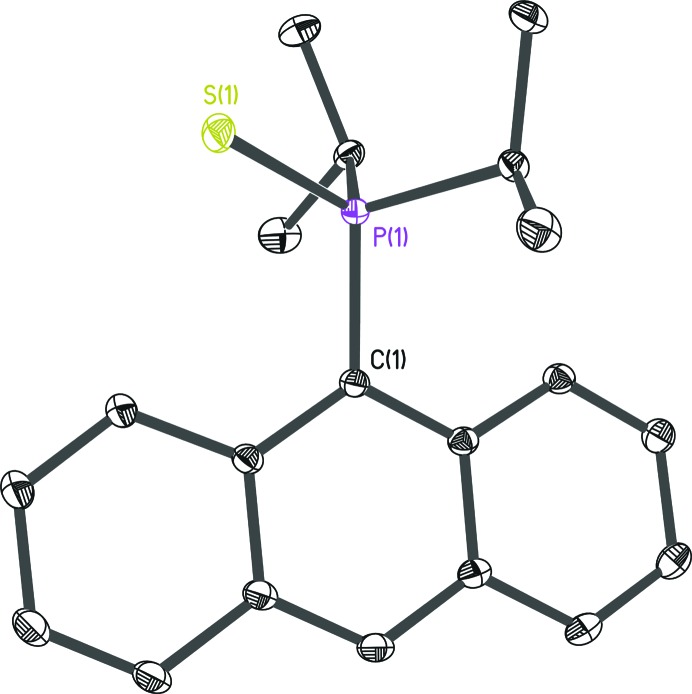
The molecular structure of **2**. Anisotropic displacement parameters are depicted at the 50% probability level. Hydrogen atoms have been omitted for clarity.

Data for structure **2** (Fig. 8 ▸) were collected on a Bruker D8 three-circle goniometer equipped with a Bruker TXS-30 Mo rotating anode with INCOATEC Helios mirror optics and an APEXII detector at 100 K. The compound crystallizes in space group *Pbca* (Krause, 2017 ▸).

**Figure 9:**
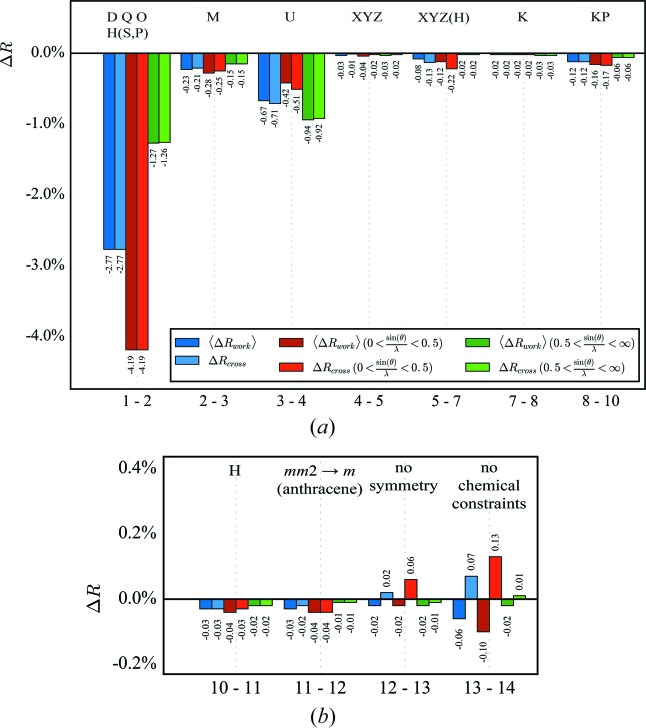
*ΔR* values for the initial refinement strategy for structure **2**. Abbreviations: M indicates monopoles, D dipoles, Q quadrupoles, O octupoles, H hexadecapoles, U *U_ij_*, K κ and KP κ′.

The local coordinate systems were defined so that the highest possible symmetry could be applied. This led to cylindrical symmetry for the H and S atoms, mirror symmetry for the methine C atoms and threefold symmetry for the methyl C atoms. For the anthracene C atoms *mm*2 symmetry was adopted. The pole populations of chemically equivalent atoms were constrained to be identical. The C-atom multipole expansion was restricted to octupoles, while for S and P atoms hexadecapoles were employed. The refinement strategy was similar to that of **1** (see the supporting information) and similar trends for the behaviour of *R*_cross_ are evident (see Fig. 9 ▸). The refinement of the multipole populations improves 〈*R*_work_〉 and *R*_cross_ significantly. Both values show an improvement that is greater from the low-order data compared with the high-order, which is in good agreement with expectations. The subsequent introduction of monopoles displays a similar picture. Next, adjustment of the displacement parameters does not show any sign of overfitting and is affected by both low- and high-order reflections. The addition of positional parameters for non-hydrogen atoms does not need adjustment to the same extent here, indicating better starting parameters. With the density modelled, a new adjustment of the H-atom positions in step 6 leads to a drop in the low-resolution *R* value, which is again in good agreement with expectations.

The refinement of the *k* parameters in step 8 stems pre­dominantly from the low-resolution data. The introduction of hexadecapoles to the C atoms also shows an improvement from the low-order data. The same is found for the expansion from *mm*2 to *m* (in the plane) symmetry for the anthracene C atoms. However, on releasing the local symmetry constraints, overfitting is indicated by a drop in 〈*R*_work_〉 and an increase in *R*_cross_, which is even more pronounced after the release of the constraints for equivalent atoms in step 14. The total data-to-parameter ratio is still 24.2, but the ratio of low-order data to the monopole or multipole populations and the κ parameter is reduced to 3.1. Ultimately, we decided to stay with the more restricted model obtained after step 12. Here, the ratio of low-order data to the monopole or multipole populations and the κ parameter is still 13.0.

### 3.3. Constraints due to crystallographic symmetry   

Atoms on special positions need constraints for the refinement. While in IAM programs like *SHELXL* these constraints are generated automatically, they need to be set manually in *XD*.

**Figure 10:**
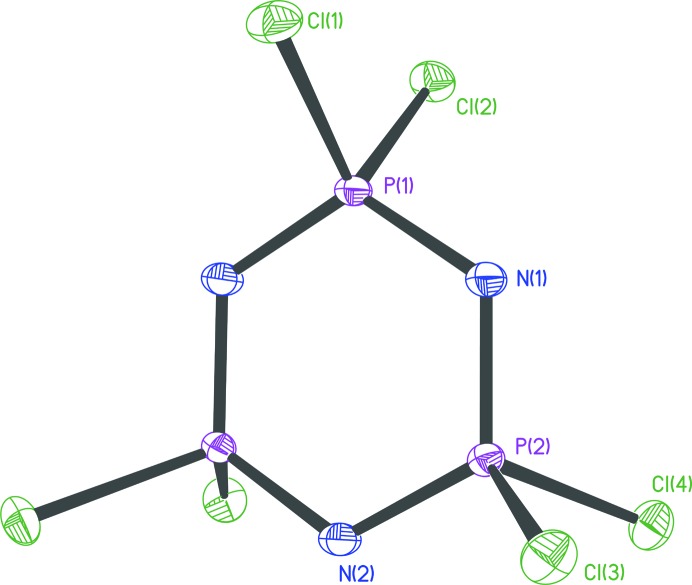
The molecular structure of **3**. Anisotropic displacement parameters are depicted at the 50% probability level.

Compound **3** (Fig. 10 ▸) crystallizes in space group *Pnma* with half a molecule in the asymmetric unit (Jancik *et al.*, 2017 ▸). Atoms P1, Cl1, Cl2 and N2 are located on a crystallographic mirror plane. Cylindrical symmetry was applied to the Cl atoms, and *mm*2 symmetry for the P and N atoms. All atoms of each element type were constrained to share the same monopole and multipole populations. Additional parameters were then added in a stepwise manner: multipole and monopole populations, and adjustment of *U*, *xyz*, κ and κ′. No refinement step indicated any overfitting (see the supporting information). However, a typical residual density distribution, indicating anharmonic motion, was present. Hence third-order Gram–Charlier coefficients, first for the Cl atoms, then for the P atoms and finally for the N atoms, were introduced. No overfitting was visible and all performed tests for a reasonable refinement of anharmonic motion were fulfilled (Jancik *et al.*, 2017 ▸). Then the local symmetry of the Cl atoms was released from cylindrical to mirror plane symmetry (data-to-parameter ratio 44.3, low-order to monopole or multipole and κ parameter 8.7). Subsequently, in a similar fashion, the symmetry was lowered for the N and P atoms from *mm*2 to *m* (data-to-parameter ratio still 41.2, low-order to monopole or multipole and κ parameter 6.4). The release of all chemical constraints for all atoms followed (data-to-parameter ratio 28.7, low-order to monopole or multipole and κ parameter 4.9). Finally, all symmetry constraints for all atoms on general positions were released (data-to-parameter ratio 24.7, low-order to monopole or multipole and κ parameter 3.7). While the release of the local symmetry contraints for the N and P atoms has only a small effect on the *R* values, the release of both chemical *and* local symmetry constraints for all atoms on general positions increases *R*_cross_, clearly indicating overfitting.

**Figure 11:**
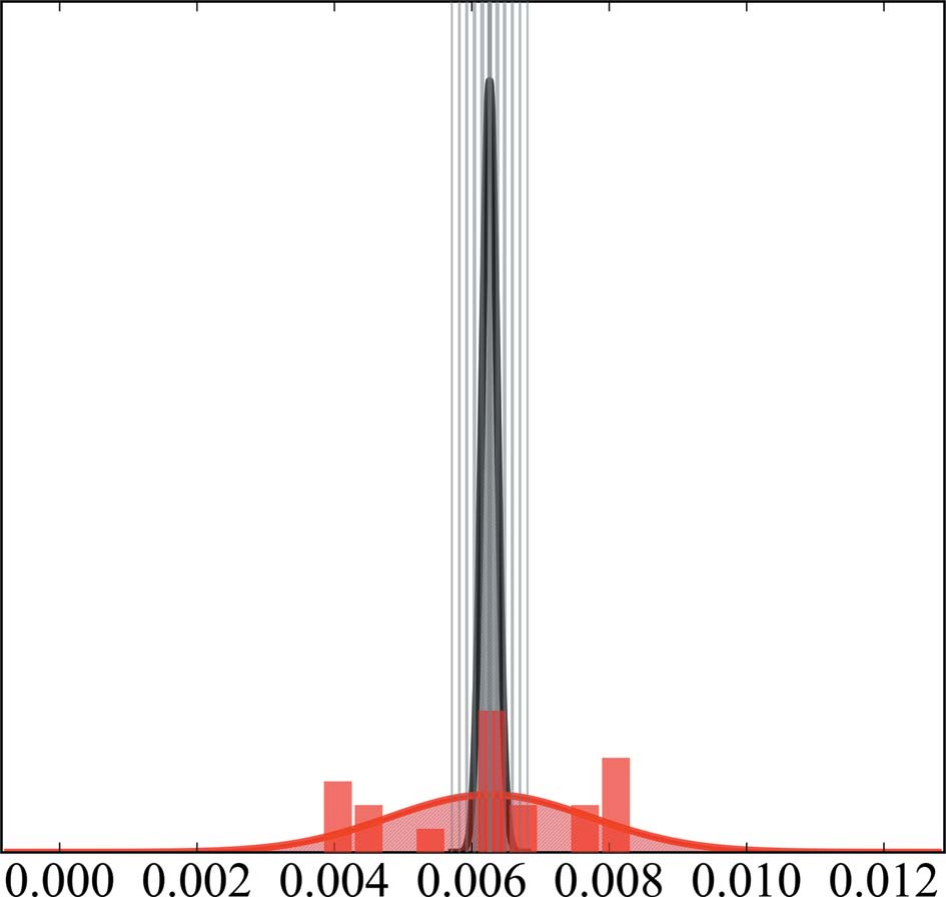
Distribution of the Gram-Charlier coefficient *C*_112_ of atom Cl1, which was refined by mistake but should be constrained to 0 due to the crystallographic mirror symmetry

In a first trial, the constraints for the Gram–Charlier co­efficients for the atoms on special positions were mistakenly left unset. The impact on Δ*R*_cross_ was imperceptible, but this error could easily be identified by inspection of the distribution of values of the *k* refinements (see Fig. 11 ▸). Of course, such an error also leads to convergence problems, but the parameter distribution is able to identify the problematic parameters quickly.

### 3.4. Outlier detection   

**Figure 12:**
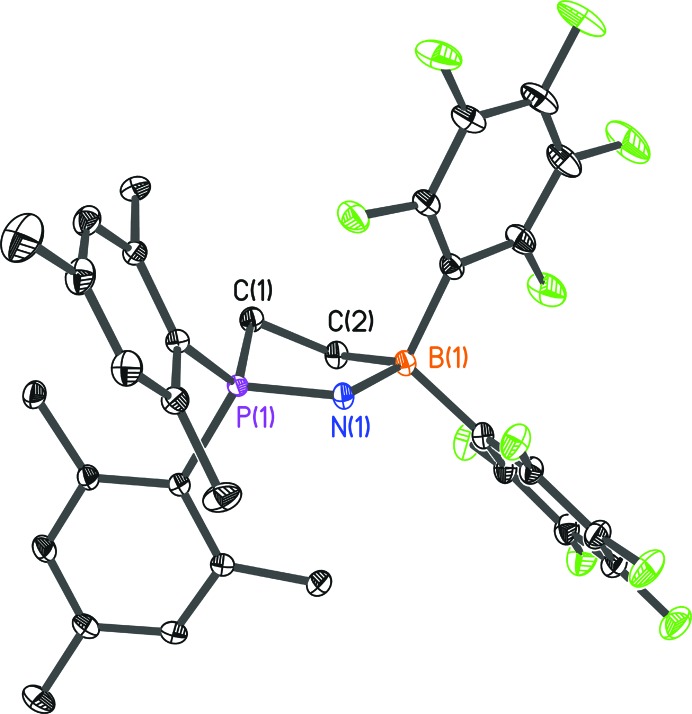
The molecular structure of **4**. Anisotropic displacement parameters are depicted at the 50% probability level. Hydrogen atoms have been omitted for clarity

Structure **4** (Stute *et al.*, 2012 ▸; Fig. 12 ▸) crystallizes in space group *P*2_1_/*n* with one molecule in the asymmetric unit.

**Figure 13:**
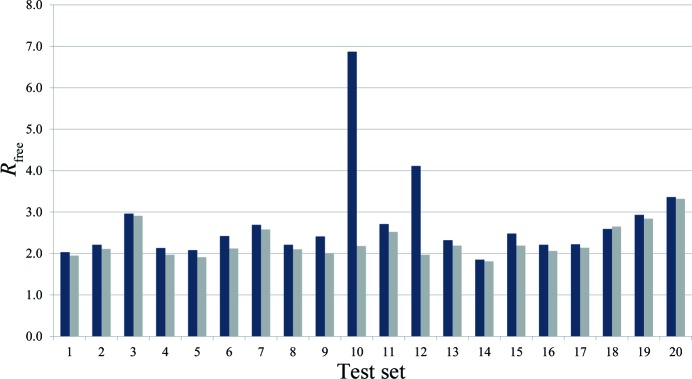
*R*_free_ values for all 20 validations sets, blue: using all data, grey: after omission of the outlying reflections.

In this structure, the check of the parameter distribution identified all outliers with |*v*_total_ − *v*_*i*_| > 3*s*_total_ (for details see the supporting information) as belonging to only three test sets. Two of these sets showed much higher *R*_free_ values than all other sets (see Fig. 13 ▸).

**Figure 14:**
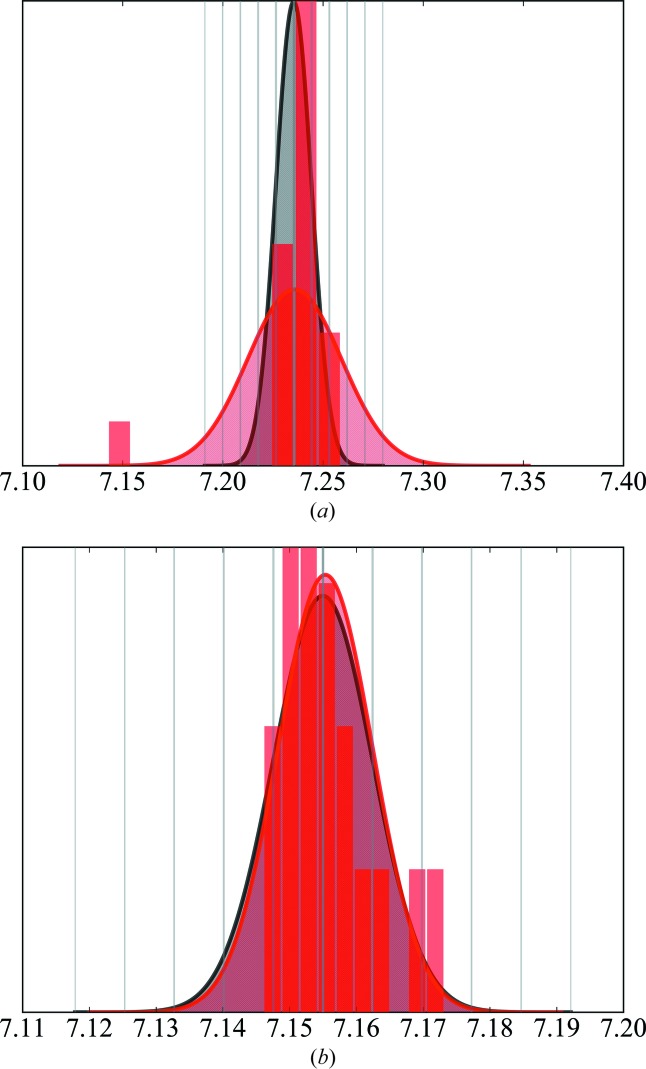
Distribution of the monopole population of atom F34, (*a*) using all data and (*b*) after omitting two outlier reflections.

Careful inspection of these two validation sets showed that in both there is one very strong low-order reflection with *F*_o_ << *F*_c_ found by inspection of the *DRK* plots (Zhurov *et al.*, 2008 ▸; Zavodnik *et al.*, 1999 ▸) (see the supporting information). These two reflections are overexposed. Strong low-order reflections have a large effect on the multipole populations, so an in­accurate determination of such reflections is highly problem­atic. Since these reflections are omitted from these two training sets, the derived parameter sets from these two training sets are not outliers but proper values. An omission of these two reflections lowers the completeness in this important low-order range but improves the model indicated by a more reasonable parameter distribution (see as an example Fig. 14 ▸ *b*).

### 3.5. Chemical constraints   

In all our tested structures, we observed the release of chemical constraints to result in serious overfitting. Recently, we published the charge-density investigation of a silylone (Niepötter *et al.*, 2014 ▸). In this structure, an Si atom is co­ordinated by two identical cAAC (cyclic alkyl amino carbene) ligands. We found that the two Si—C bonds differ significantly in both length and ellipticity. Therefore, we anticipated that the release of chemical constraints is necessary for a proper modelling of this structure (for details of the *R*_cross_ procedure see the supporting information). However, unexpectedly, only the release of the local symmetry constraints of the Si atom proved necessary to describe the differences in the two Si—C bonds. In contrast, the release of the chemical constraints of the two complete carbene ligands showed severe signs of overfitting, further emphasizing chemical constraints to be important in stabilizing a multipole refinement, at least for data sets to a resolution of 0.5–0.4 Å.

## 4. Conclusions   

The presented method of cross-validation is a valid tool in multipole refinement. Although the number of data in a high-resolution data set is high enough to achieve a global data-to-parameter ratio larger than 1:10 or even 1:20, even when all possible multipole parameters up to the hexadecapole level are refined, it has to be considered that not all reflections contribute equally to all multipole parameters. The information about the valence density is mainly gained from the relatively few low-order reflections. Therefore, the simple global data-to-parameter rule of thumb is not sufficient to decide on the advisable number of parameters. In contrast with the well established *R*_free_ in macromolecular crystallography (Brünger, 1992 ▸, 1997 ▸) or the new *R*_complete_ concept (Luebben & Gruene, 2015 ▸), this method is not interested in absolute *R*_cross_ values but in the progress of *R*_cross_ along the refinement strategy. For a reasonable refinement, not only must the normal *R* values decrease but also the *R*_cross_ value. Investigating several structures with this approach, we noticed the following general aspects:

(i) It helps to start with very high symmetry defined only by next neighbours.

(ii) Lowering these symmetry constraints often does not improve the model significantly. Normally it is sufficient to release these constraints for only a few atoms. Any un­expected large drop in *R*_cross_ is suspicious and helps to detect mistakes in the definition of the local coordinate system or the assumed local symmetry.

(iii) Release of all chemical constraints clearly causes overfitting in all investigated structures.

(iv) Unfortunately, *XD* does not offer restraints. However, using restraints instead of constraints could be a further improvement of the model (Paul *et al.*, 2011 ▸; Zarychta *et al.*, 2011 ▸). A protocol for oriented local atomic axes is explained in detail by Domagała & Jelsch (2008 ▸).

Additional to validation of the refinement strategy, analysis of the parameter distribution provides access to further causes of defects. Overlooking the necessary constraints required by crystallographic symmetry can easily be prevented. Inaccurately determined strong low-order reflections that bias the derived parameters can be easily identified.

The *k* different refinements also provide a distribution of the topological properties, leading to a rough estimate for the standard deviations of these parameters.

## Supplementary Material

Crystal structure: contains datablock(s) 1_shelx, 1_xd, 2_shelx, 2_xd. DOI: 10.1107/S2052252517005103/lc5072sup1.cif

Structure factors: contains datablock(s) 1. DOI: 10.1107/S2052252517005103/lc50721sup2.hkl

Structure factors: contains datablock(s) 2. DOI: 10.1107/S2052252517005103/lc50722sup3.hkl

Supplementary tables and figures. DOI: 10.1107/S2052252517005103/lc5072sup4.pdf

CCDC references: 1545979, 1545980, 1545981

## Figures and Tables

**Table 1:** Properties (ρ in e Å^−3^ and ∇^2^ρ in e Å^−5^) at the bond critical points of the B—C and B—N bonds, comparing the final refinement strategy (second row) with the initial strategy without local symmetry or chemical constraints (first row)

Bond	Property	*v*_total_	*s*_total_	*v*_mean_	*s*_mean_	|*v*_mean_ − *v*_total_|/*s*_mean_	*s*_mean_/*s*_total_
C1—B1	ρ	1.139	0.012	1.141	0.007	0.3	0.6
		1.154	0.012	1.156	0.006	0.2	0.5
	∇^2^ρ	−5.86	0.06	−6.0	0.6	0.2	11.2
		−7.16	0.06	−7.2	0.5	0.2	9.7
	∊	0.03		0.042	0.019	0.5	
		0.05		0.055	0.013	0.4	
C21—B1	ρ	1.023	0.011	1.024	0.010	0.1	0.9
		1.057	0.009	1.059	0.005	0.5	0.5
	∇^2^ρ	−3.01	0.05	−3.1	0.6	0.1	12.4
		−5.68	0.04	−5.8	0.4	0.3	9.2
	∊	0.11		0.11	0.02	0.2	
		0.09		0.094	0.009	0.5	
C31—B1	ρ	1.050	0.011	1.049	0.007	0.1	0.7
		1.055	0.010	1.055	0.005	0.1	0.5
	∇^2^ρ	−7.47	0.05	−7.5	0.4	0.1	7.6
		−6.20	0.04	−6.2	0.4	0.1	9.2
	∊	0.18		0.18	0.02	0.2	
		0.15		0.15	0.02	0.2	
N1—B1	ρ	0.779	0.011	0.790	0.005	0.6	0.5
		0.747	0.011	0.749	0.005	0.4	0.4
	∇^2^ρ	−0.94	0.04	−1.1	0.3	0.4	7.1
		1.86	0.05	1.8	0.4	0.2	7.9
	∊	0.48		0.49	0.06	0.2	
		0.65		0.66	0.06	0.1	

## References

[bb1] Allen, F. H. & Bruno, I. J. (2010). *Acta Cryst.* B**66**, 380–386. 10.1107/S0108768110012048 20484809

[bb2] Bader, R. F. W. (1990). *Atoms In Molecules: A Quantum Theory.* Oxford; New York: Clarendon Press.

[bb3] Brünger, A. T. (1992). *Nature*, **355**, 472–475. 10.1038/355472a0 18481394

[bb4] Brünger, A. T. (1997). *Methods Enzymol.* **277**, 366–396. 10.1016/s0076-6879(97)77021-6 18488318

[bb5] Cochran, W. G. & Wishart, J. (1934). *Math. Proc. Cambridge Philos. Soc.* **30**, 178–191.

[bb6] Coppens, P. (2005). *Angew. Chem. Int. Ed.* **44**, 6810–6811. 10.1002/anie.200501734 16187393

[bb7] Domagała, S. & Jelsch, C. (2008). *J. Appl. Cryst.* **41**, 1140–1149.

[bb8] Domagała, S., Kosc, K., Robinson, S. W., Haynes, D. A. & Woźniak, K. (2014). *Cryst. Growth Des.* **14**, 4834–4848.

[bb9] Dos Santos, L. H. R., Lanza, A., Barton, A. M., Brambleby, J., Blackmore, W. J. A., Goddard, P. A., Xiao, F., Williams, R. C., Lancaster, T., Pratt, F. L., Blundell, S. J., Singleton, J., Manson, J. L. & Macchi, P. (2016). *J. Am. Chem. Soc.* **138**, 2280–2291. 10.1021/jacs.5b12817 26811927

[bb10] Hansen, N. K. & Coppens, P. (1978). *Acta Cryst.* A**34**, 909–921.

[bb11] Herbst-Irmer, R., Henn, J., Holstein, J. J., Hübschle, C. B., Dittrich, B., Stern, D., Kratzert, D. & Stalke, D. (2013). *J. Phys. Chem. A*, **117**, 633–641. 10.1021/jp309985e 23241030

[bb12] Hübschle, C. B. & Dittrich, B. (2011). *J. Appl. Cryst.* **44**, 238–240. 10.1107/S0021889810042482 PMC3253731 22477783

[bb13] Jancik, V., Cortés-Guzmán, F., Herbst-Irmer, R. & Martínez-Otero, D. (2017). *Chem. Eur. J.* DOI:10.1002/chem.201700411. 10.1002/chem.201700411 28135404

[bb14] Kamiński, R., Domagała, S., Jarzembska, K. N., Hoser, A. A., Sanjuan-Szklarz, W. F., Gutmann, M. J., Makal, A., Malińska, M., Bąk, J. M. & Woźniak, K. (2014). *Acta Cryst.* A**70**, 72–91. 10.1107/S2053273313028313 24419172

[bb15] Krause, L. (2017). PhD thesis, Georg-August-Universität, Göttingen, Germany.

[bb16] Kuhs, W. F. (1992). *Acta Cryst.* A**48**, 80–98.

[bb17] Luebben, J. & Gruene, T. (2015). *Proc. Natl Acad. Sci. USA*, **112**, 8999–9003. 10.1073/pnas.1502136112 PMC4517205 26150515

[bb18] Meindl, K. & Henn, J. (2008). *Acta Cryst.* A**64**, 404–418. 10.1107/S0108767308006879 18421130

[bb19] Niepötter, B., Herbst-Irmer, R., Kratzert, D., Samuel, P. P., Mondal, K. C., Roesky, H. W., Jerabek, P., Frenking, G. & Stalke, D. (2014). *Angew. Chem. Int. Ed.* **53**, 2766–2770. 10.1002/anie.201308609 24481811

[bb20] Paul, A., Kubicki, M., Jelsch, C., Durand, P. & Lecomte, C. (2011). *Acta Cryst.* B**67**, 365–378. 10.1107/S0108768111022683 21775815

[bb21] Poulain, A., Wenger, E., Durand, P., Jarzembska, K. N., Kamiński, R., Fertey, P., Kubicki, M. & Lecomte, C. (2014). *IUCrJ*, **1**, 110–118. 10.1107/S2052252514002838 PMC4062092 25075327

[bb22] Schwendemann, S., Fröhlich, R., Kehr, G. & Erker, G. (2011). *Chem. Sci.* **2**, 1842–1849.

[bb23] Shapiro, S. S. & Wilk, M. B. (1965). *Biometrika*, **52**, 591–611.

[bb24] Sheldrick, G. M. (2008). *Acta Cryst.* A**64**, 112–122. 10.1107/S0108767307043930 18156677

[bb25] Sheldrick, G. M. (2015). *Acta Cryst.* C**71**, 3–8.

[bb26] Stute, A., Heletta, L., Fröhlich, R., Daniliuc, C. G., Kehr, G. & Erker, G. (2012). *Chem. Commun.* **48**, 11739–11741. 10.1039/c2cc36782c 23111350

[bb27] Tickle, I. J., Laskowski, R. A. & Moss, D. S. (2000). *Acta Cryst.* D**56**, 442–450. 10.1107/s0907444999016868 10739917

[bb28] Volkov, A., Macchi, P., Farrugia, L. J., Gatti, C., Mallinson, P. R., Richter, T. & Koritsanszky, T. (2006). *XD2006*, Revision 5.34. University of New York at Buffalo, New York, USA.

[bb29] Zarychta, B., Zaleski, J., Kyzioł, J., Daszkiewicz, Z. & Jelsch, C. (2011). *Acta Cryst.* B**67**, 250–262. 10.1107/S0108768111013140 21586833

[bb30] Zavodnik, V., Stash, A., Tsirelson, V., de Vries, R. & Feil, D. (1999). *Acta Cryst.* B**55**, 45–54. 10.1107/s0108768198005746 10927338

[bb31] Zhurov, V. V., Zhurova, E. A. & Pinkerton, A. A. (2008). *J. Appl. Cryst.* **41**, 340–349.

