# Supplementary material for: Validation of experimental charge-density refinement strategies: when do we overfit?
Source: IUCrJ. 2017 May 24;4(Pt 4):420–30. doi: 10.1107/S2052252517005103 (PMC5571805; doi:10.1107/S2052252517005103)

IUCrJ

Volume 4 (2017)

Supporting information for article:

Validation of experimental charge density refinement strategies: When do we overfit?

Lennard Krause, Benedikt Niepötter, Christian J. Schürmann, Dietmar Stalke and Regine Herbst-Irmer

Supporting information

Validation of experimental charge density refinement strategies: When do we overfit?

Lennard Krause, Benedikt Niepötter, Christian J. Schürmann, Dietmar Stalke and Regine Herbst-Irmer*

Universität Göttingen, Institut für Anorganische Chemie, Tammannstraße 4, Göttingen, 37077, Germany

Correspondence email: rherbst@shelx.uni-ac.gwdg.de

S1. Structure 1

S1.1. Local coordinate system for the first strategy

ATOM	ATOM0	AX1	ATOM1	ATOM2	AX2	Symm	CHEMCON	κ set
F(22)	C(22)	Z	F(22)	C(21)	Y	_cy	-	1
F(23)	C(23)	Z	F(23)	C(22)	Y	_cy	-	1
F(24)	C(24)	Z	F(24)	C(23)	Y	_cy	-	1
F(25)	C(25)	Z	F(25)	C(26)	Y	_cy	F(23)	1
F(26)	C(26)	Z	F(26)	C(21)	Y	_cy	F(22)	1
F(32)	C(32)	Z	F(32)	C(31)	Y	_cy	F(22)	1
F(33)	C(33)	Z	F(33)	C(32)	Y	_cy	F(23)	1
F(34)	C(34)	Z	F(34)	C(33)	Y	_cy	F(24)	1
F(35)	C(35)	Z	F(35)	C(36)	Y	_cy	F(23)	1
F(36)	C(36)	Z	F(36)	C(31)	Y	_cy	F(22)	1
N(1)	B(1)	X	N(1)	C(2)	Y		-	2
C(1)	B(1)	X	C(1)	C(2)	Y		-	3
C(2)	N(1)	Z	C(2)	C(1)	Y		-	4
C(3)	N(1)	X	C(3)	C(4)	Y	_mz	-	5
C(4)	C(3)	Z	C(4)	H(4A)	Y	_3m	-	6
C(5)	N(1)	X	C(5)	C(6)	Y	_mz	C(3)	5
C(6)	C(5)	Z	C(6)	H(6B)	Y	_3m	C(4)	6
C(11)	C(12)	X	C(11)	C(16)	Y	_mz	-	7
C(12)	C(13)	X	C(12)	C(11)	Y	_mz	-	7
C(13)	C(14)	X	C(13)	C(12)	Y	_mz	-	7
C(14)	H(14)	Z	C(14)	C(15)	Y	_mm2	-	7
C(15)	C(14)	X	C(15)	C(16)	Y	_mz	C(13)	7
C(16)	C(15)	X	C(16)	C(11)	Y	_mz	C(12)	7
C(21)	C(22)	X	C(21)	C(26)	Y	_mz	-	8
C(22)	C(23)	X	C(22)	C(21)	Y	_mz	-	8
C(23)	C(24)	X	C(23)	C(22)	Y	_mz	-	8
C(24)	F(24)	Z	C(24)	C(25)	Y	_mm2	-	8
C(25)	C(24)	X	C(25)	C(26)	Y	_mz	C(23)	8
C(26)	C(25)	X	C(26)	C(21)	Y	_mz	C(22)	8
C(31)	C(32)	X	C(31)	C(36)	Y	_mz	C(21)	8
C(32)	C(33)	X	C(32)	C(31)	Y	_mz	C(22)	8
C(33)	C(34)	X	C(33)	C(32)	Y	_mz	C(23)	8
C(34)	F(34)	Z	C(34)	C(35)	Y	_mm2	C(24)	8
C(35)	C(34)	X	C(35)	C(36)	Y	_mz	C(23)	8

C(36)	C(35)	X	C(36)	C(31)	Y	_mz	C(22)	8
B(1)	N(1)	X	B(1)	C(1)	Y		-	9
H(1A)	C(1)	Z	H(1A)	H(1B)	Y	_cy	-	10
H(1B)	C(1)	Z	H(1B)	H(1A)	Y	_cy	H(1A)	10
H(2)	C(2)	Z	H(2)	C(11)	Y	_cy	-	10
H(3A)	C(3)	Z	H(3A)	H(3B)	Y	_cy	-	10
H(3B)	C(3)	Z	H(3B)	H(3A)	Y	_cy	H(3A)	10
H(4A)	C(4)	Z	H(4A)	H(4B)	Y	_cy	-	10
H(4B)	C(4)	Z	H(4B)	H(4C)	Y	_cy	H(4A)	10
H(4C)	C(4)	Z	H(4C)	H(4A)	Y	_cy	H(4A)	10
H(5A)	C(5)	Z	H(5A)	H(5B)	Y	_cy	H(3A)	10
H(5B)	C(5)	Z	H(5B)	H(5A)	Y	_cy	H(3A)	10
H(6A)	C(6)	Z	H(6A)	H(6B)	Y	_cy	H(4A)	10
H(6B)	C(6)	Z	H(6B)	H(6C)	Y	_cy	H(4A)	10
H(6C)	C(6)	Z	H(6C)	H(6A)	Y	_cy	H(4A)	10
H(12)	C(12)	Z	H(12)	C(13)	Y	_cy	-	10
H(13)	C(13)	Z	H(13)	C(14)	Y	_cy	H(12)	10
H(14)	C(14)	Z	H(14)	C(15)	Y	_cy	H(12)	10
H(15)	C(15)	Z	H(15)	C(16)	Y	_cy	H(12)	10
H(16)	C(16)	Z	H(16)	C(11)	Y	_cy	H(12)	10

S1.2. First *XD* refinement strategy

Abbreviations: M: monopoles; D: dipoles; Q: quadrupoles; O: octupoles; H: hexadecapoles, U: Uij, k: kappa, C(atom name): Gram Charlier 3rd order, nosym: no local symmetry constraints, nocon: no chemical constraints.

The scale factor is refined in every step but only mentioned in the first.

The new added parameter is marked in red while the last refinement step with model improvement and no overfitting is marked in green.

Step	Parameter	# param.	# data	*d*/*p*	d_l/p_m	σ cut off	Res. [Å^{-1}]	$R(F^2)$
1	Scale factor	1	15393	15393	None	3	1.00	4.37
2	DOQH	234	15393	65.8	9.59	3	1.00	1.58
3	DOQHk	243	15393	63.3	9.24	3	1.00	1.55
4	MDOQHk	264	15393	58.3	8.47	3	1.00	1.45
5	MDOQHkU	480	15393	32.1	8.47	3	1.00	1.39
6	MDOQHkUxyzk	588	15393	26.2	8.47	3	1.00	1.27
7	xyz(H)	55	2235	40.6	None	3	0.50	0.86

8	MDOQHUxyzk [xyz(H)]	588	15393	26.2	8.47	3	1.00	1.11
9	k'	10	15393	1539.3	None	3	1.00	1.10
10	MDOQHUxyzk [k']	588	15393	26.2	8.47	3	1.00	1.09
11	MDOQHUxyzk [sig obs = 0]	588	17600	29.9	8.52	0	1.00	1.14
12	MDOQHUxyzk [res. 1.06]	588	20737	35.3	8.52	0	1.06	1.22
13	MDOQHUxyzk (F only m)	618	20737	33.7	7.65	0	1.06	1.21
14	MDOQHUxyzk (nosym)	768	20737	27.0	5.07	0	1.06	1.18
15	MDOQHUxyzk (nosym nocon)	1243	20737	16.7	2.45	0	1.06	1.08

d/p: data to parameter ratio, d_l/p_m: low-resolution ($\sin(\theta)/\lambda$) < 0.5 Å^{-1}) data to mono-, multipole ($\kappa^{(\prime)}$) parameter ratio

S1.3. Anharmonic refinement of F24, F33, F34, F35

S1.3.1. First Strategy

Step	Parameter	# param.	# data	d/p	d_l/p_m	σ cut off	Res. [Å^{-1}]	$R(F^2)$
1-12	As before	-	-	-	-	-	-	-
13	MDOQHUxyzk C(F33-F35)	618	20737	33.6	8.5	0	1.06	1.19
14	MDOQHUxyzk C(F33-F35, F24)	628	20737	33.0	8.5	0	1.06	1.19

d/p: data to parameter ratio, d_l/p_m: low-resolution ($\sin(\theta)/\lambda$) < 0.5 Å^{-1}) data to mono-, multipole ($\kappa^{(\prime)}$) parameter ratio

S1.3.2. Residual density before and after anharmonic refinement

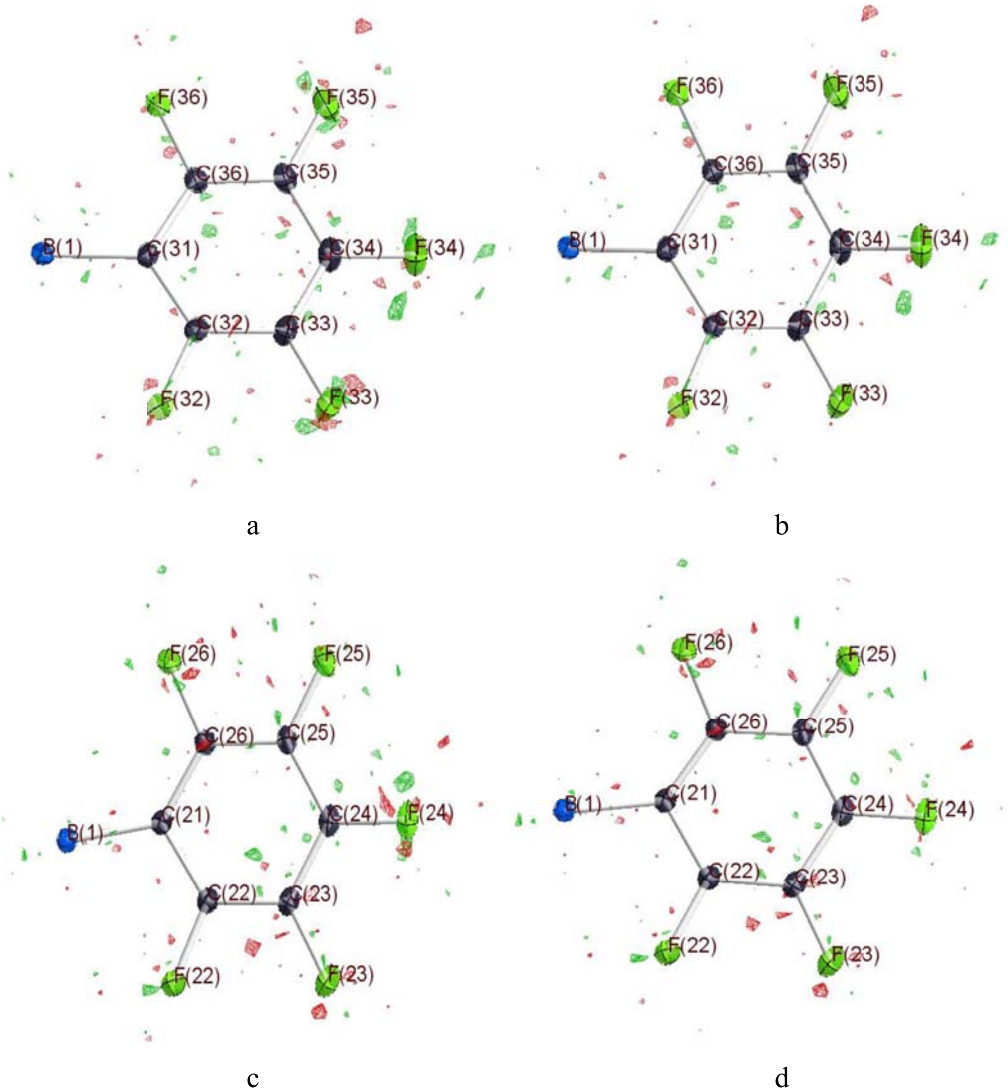

Figure S1 Residual density isosurfaces. The green density is positive and red negative, isosurface level ±0.062 e Å^{-3}. (a) and (c) before and (b) and (d) after anharmonic refinement

S1.3.3. Kuhs' rule

The table below shows the minimum data resolution required for meaningful refinement of anharmonic thermal parameters (3rd order Gram-Charlier coefficients), for each anisotropic atom. See - Kuhs, W. F. (1992) Acta Cryst A48, 80-98.

Atom	Principal M.D.A.'s (A)			Min. resolution sin(θ)/λ
F(24)	0.192	0.159	0.118	0.84
F(33)	0.213	0.173	0.126	0.78
F(34)	0.231	0.167	0.122	0.77
F(35)	0.227	0.172	0.126	0.76

S1.3.4. Extreme displacements in the map from the equilibrium position of atom F(24), F(33) to F(35) in Angstroms along reciprocal axial directions. deltaX/Y/Z = -0.80 to 0.80

Atom	F(24)	F(33)	F(34)	F(35)
Minimum PDF value	-105.38	-0.87	-52.24	-0.34
Maximum PDF value	75920.52	58693.58	57712.98	55385.62
Total integrated negative probability [%]	-0.046	0.000	-0.026	0.000
Integrated volume for negative probability [Å^3]	1.268	0.412	0.975	0.443
Total integrated positive probability [%]	100.045	99.988	100.013	99.997
Integrated volume for positive probability [Å^3]	3.071	3.928	3.365	3.897

S1.4. Anharmonic refinement of F33, F34, F35

S1.4.1. Strategy

Step	Parameter	# param.	# data	*d*/*p*	d_l/p_m	σ cut off	Res. [Å^{-1}]	R
1-12	As before	-	-	-		-	-	-
13	MDOQHUxyzk C(F33-F35)	618	20737	33.6	8.5	0	1.06	1.19
14	MDOQHUxyzk C(F33-F35) (F only m)	648	20737	32.0	7.7	0	1.06	1.19
15	MDOQHUxyzk C(F33-F35) (nosym)	798	20737	26.0	5.1	0	1.06	1.16
16	MDOQHUxyzk C(F33-F35) (nosym noconn)	1273	20737	16.3	2.5	0	1.06	1.07

d/*p*: data to parameter ratio, d_l/p_m: low-resolution ($\sin(\theta)/\lambda$) < 0.5 Å^{-1}) data to mono-, multipole ($\kappa^{(')}$) parameter ratio

S1.4.2. Cross-validation

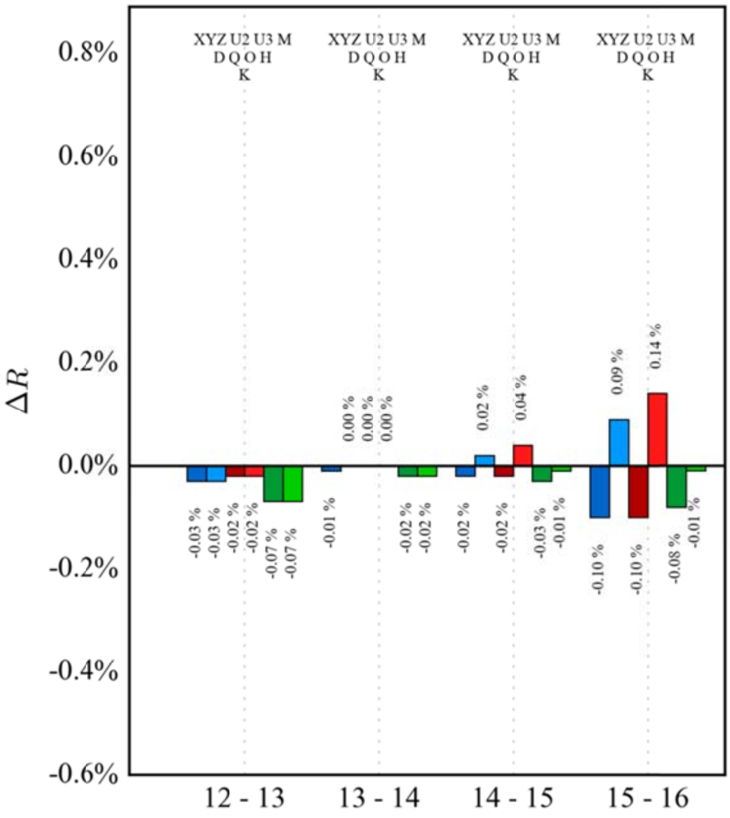

S1.4.3. Extreme displacements in the map from the equilibrium position of atom F(33) to F(35) in Angstroms along reciprocal axial directions. deltaX/Y/Z = -0.80 to 0.80 F(24) refined harmonically

Atom	F(33)	F(34)	F(35)
Smallest PDF value	-0.86	-1.06	-0.34
Largest PDF value	58687.32	57608.65	55384.11
Total integrated negative probability [%]	0.000	0.000	0.000
Integrated volume for negative probability [Å^3]	0.425	0.579	0.395
Total integrated positive probability [%]	99.988	99.988	99.997
Integrated volume for positive probability [Å^3]	3.915	3.761	3.945

S1.4.4. Refined Gram Charlier parameters, in bold values larger than 3 σ

Atom	Cijk	value	s.u.	Atom	Cijk	value	s.u.
**F33**	C111	-0.000001	0.000005		C113	**-0.000109**	**0.000007**
	C222	**0.000012**	**0.000003**		C133	**-0.000138**	**0.000014**
	C333	0.000100	0.000051		C223	-0.000010	0.000005
	C112	**-0.000007**	**0.000002**		C233	**0.000038**	**0.000013**
	C122	**-0.000014**	**0.000002**		C123	**0.000043**	**0.000004**
	C113	**-0.000103**	**0.000006**	**F35**	C111	-0.000005	0.000004
	C133	**-0.000107**	**0.000014**		C222	**-0.000032**	**0.000004**
	C223	**0.000022**	**0.000005**		C333	**-0.000369**	**0.000072**
	C233	**0.000037**	**0.000012**		C112	**0.000008**	**0.000002**
	C123	**-0.000035**	**0.000004**		C122	**0.000012**	**0.000002**
**F34**	C111	-0.000008	0.000005		C113	-0.000011	0.000006
	C222	**-0.000013**	**0.000004**		C133	**-0.000082**	**0.000016**
	C333	**-0.000278**	**0.000056**		C223	-0.000007	0.000006
	C112	-0.000007	0.000003		C233	**0.000146**	**0.000015**
	C122	0.000003	0.000002		C123	**0.000050**	**0.000004**

S1.4.5. Parameter distribution of outliers

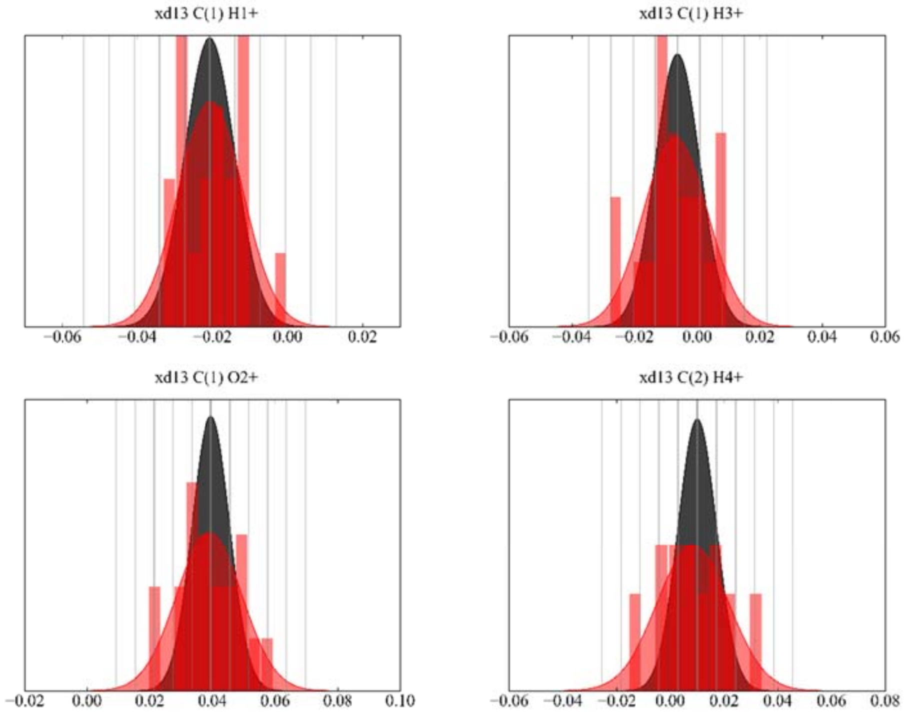

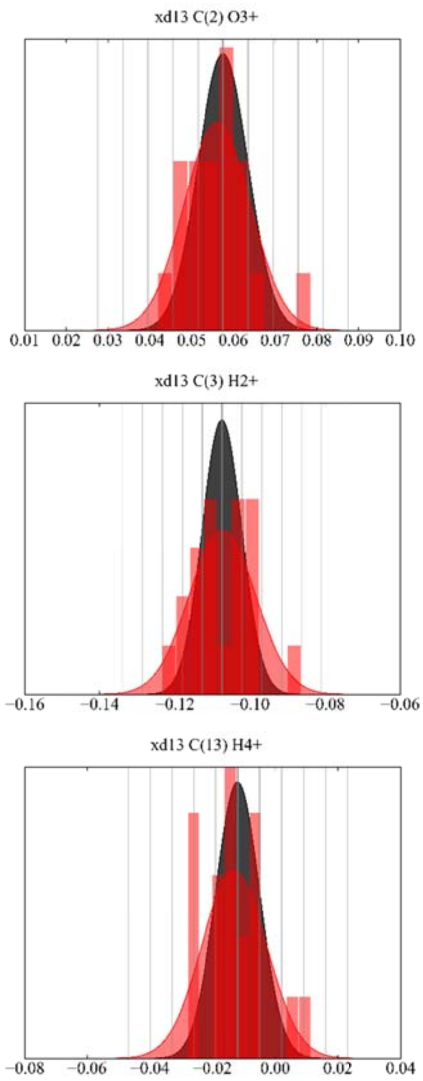

S1.5. Final Strategy: Increasing the local symmetry and checking the hexadecapole populations

S1.5.1. New local coordinate system or higher local symmetry for the following atoms

ATOM	ATOM0	AX1	ATOM1	ATOM2	AX2	Symm	CHEMCON	κ set
N(1)	B(1)	X	N(1)	C(2)	Y	_mz		2
C(1)	B(1)	X	C(1)	C(2)	Y	_mz		3
C(11)	C(14)	Z	C(11)	C(16)	Y	_mm2		7
C(12)	C(15)	Z	C(12)	C(11)	Y	_mm2		7
C(13)	C(16)	Z	C(13)	C(12)	Y	_mm2		7
C(14)	C(11)	Z	C(14)	C(15)	Y	_mm2		7
C(15)	C(12)	Z	C(15)	C(16)	Y	_mm2	C(13)	7
C(16)	C(13)	Z	C(16)	C(11)	Y	_mm2	C(12)	7

C(21)	C(24)	Z	C(21)	C(26)	Y	_mm2		8
C(22)	C(25)	Z	C(22)	C(21)	Y	_mm2		8
C(23)	C(26)	Z	C(23)	C(22)	Y	_mm2		8
C(24)	C(21)	Z	C(24)	C(25)	Y	_mm2		8
C(25)	C(22)	Z	C(25)	C(26)	Y	_mm2	C(23)	8
C(26)	C(23)	Z	C(26)	C(21)	Y	_mm2	C(22)	8
C(31)	C(34)	Z	C(31)	C(36)	Y	_mm2	C(21)	8
C(32)	C(35)	Z	C(32)	C(31)	Y	_mm2	C(22)	8
C(33)	C(36)	Z	C(33)	C(32)	Y	_mm2	C(23)	8
C(34)	C(31)	Z	C(34)	C(35)	Y	_mm2	C(24)	8
C(35)	C(32)	Z	C(35)	C(36)	Y	_mm2	C(23)	8
C(36)	C(33)	Z	C(36)	C(31)	Y	_mm2	C(22)	8
B(1)	N(1)	X	B(1)	C(1)	Y	_mz		9

S1.5.2. *XD* Strategy

Step	Parameter	# param.	# data	d/p	d_l/p_m	σ cut off	Res. [Å^{-1}]	$R(F^2)$
1	Scale factor	1	15393	15393.0	None	3	1.00	4.37
2	DOQ	110	15393	65.8	20.50	3	1.00	1.69
3	DOQk	119	15393	63.3	18.94	3	1.00	1.66
4	MDOQk	140	15393	58.3	15.96	3	1.00	1.53
5	MDOQkU	356	15393	32.1	15.96	3	1.00	1.47
6	MDOQUxyzk	464	15393	33.2	15.96	3	1.00	1.37
7	xyz(H)	55	2235	40.6	None	3	0.50	0.96
8	MDOQUxyzk [xyz(H)]	464	15393	33.2	15.96	3	1.00	1.21
9	k’	10	15393	1539.3	None	3	1.00	1.20
10	MDOQUxyzk [k’]	464	15393	33.2	15.96	3	1.00	1.19
11	MDOQUxyzk [sigob=0]	464	17600	37.9	16.06	0	1.00	1.25
12	MDOQUxyzk [res=1.06]	464	20737	44.7	16.06	0	1.06	1.32
13	MDOQH(without C1-C3, B1, N1)Uxyzk	498	20737	41.6	12.93	0	1.06	1.29
14	MDOQHUxyzk	522	20737	39.7	11.36	0	1.06	1.26
15	MDOQHUxyzk C(F33-F35)	552	20737	37.6	11.36	0	1.06	1.23
16	MDOQHUxyzk C(F33-F35) (C1,B1,N1 nosym)	582	20737	35.6	9.86	0	1.06	1.22
17	MDOQHUxyzk C(F33-F35), (C1,B1;N1 nosym), (Ph only m)	618	20737	33.6	8.52	0	1.06	1.20
18	MDOQHUxyzk (Ph only m) (C1,B1;N1 nosym), C(F33-F35), F m	648	20737	32.0	7.65	0	1.06	1.19
19	MDOQHUxyzk, C(F33-F35) nosym	798	20737	26.0	5.07	0	1.06	1.16
20	MDOQHUxyzk C(F33-F35) nosym nocon	1254	20737	16.5	2.45	0	1.06	1.07

d/p: data to parameter ratio, d_l/p_m: low-resolution ($\sin(\theta)/\lambda$) < 0.5 Å^{-1}) data to mono-, multipole ($\kappa^{(')}$) parameter ratio

S1.5.3. Cross-validation

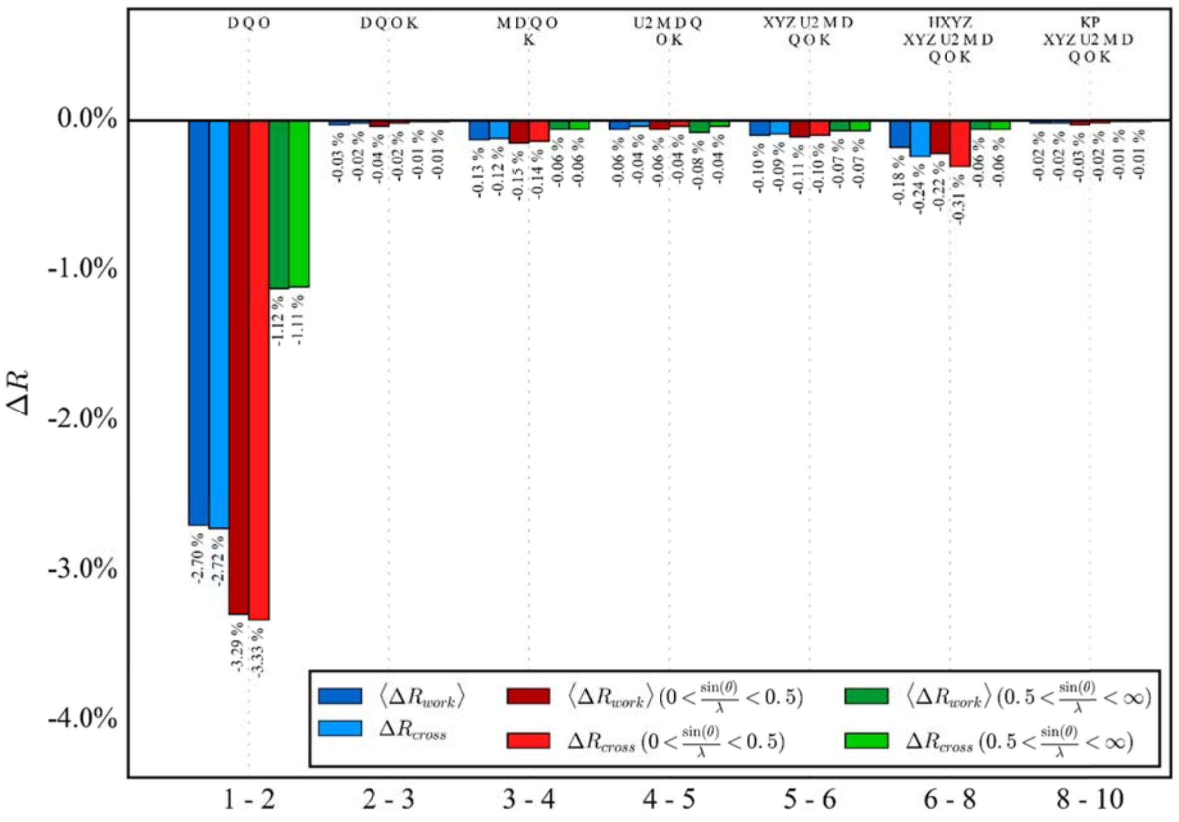

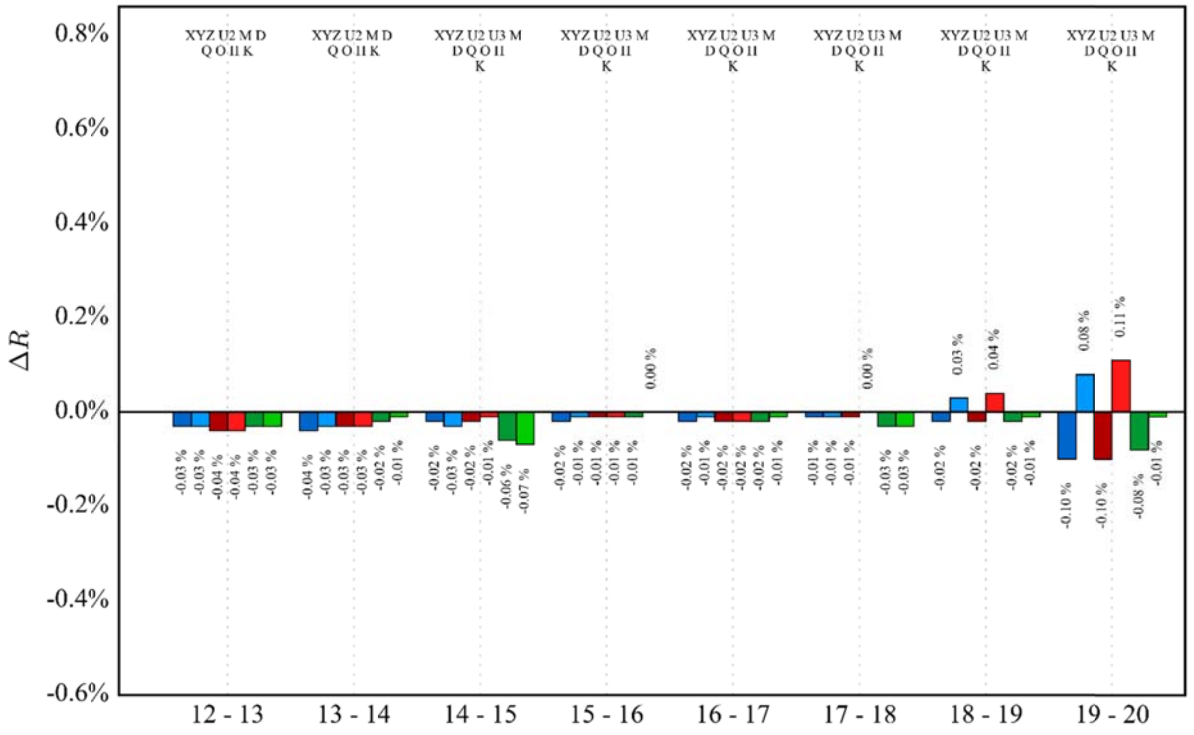

S1.5.4. Parameter distribution of outliers

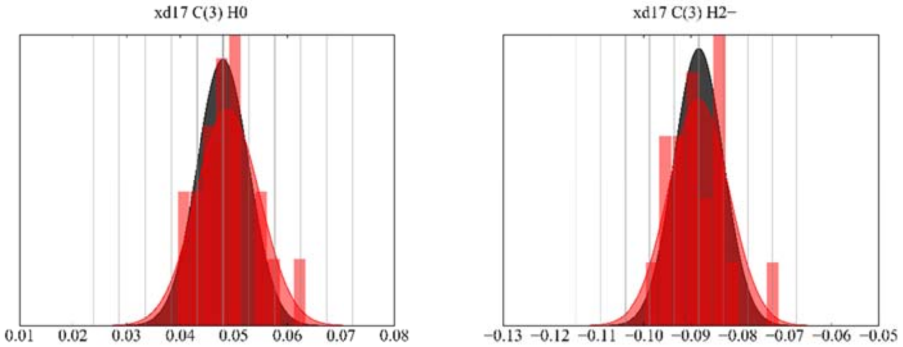

S1.5.5. ρ, ∇2ρ and ε at the BCP of the B-N bond for the last three steps of the first refinement strategy

Refinement step	Property	v_{total}	s_{total}	v_{mean}	s_{mean}	$\frac{\lvert v_{mean} - v_{total}\rvert}{s_{mean}}$	$\frac{s_{mean}}{s_{total}}$
13	ρ	0.751	0.01	0.753	0.005	0.4	0.5
14	ρ	0.75	0.01	0.751	0.005	0.3	0.5
15	ρ	0.787	0.01	0.790	0.005	0.6	0.5
13	$\nabla^2\rho$	1.08	0.04	1.0	0.3	0.2	7.9
14	$\nabla^2\rho$	1.17	0.04	1.1	0.3	0.2	8.0
15	$\nabla^2\rho$	-0.94	0.04	-1.1	0.3	0.4	7.1
13	ε	0.56	-	0.56	0.05	0.1	-
14	ε	0.51	-	0.50	0.05	0.1	-
15	ε	0.48	-	0.49	0.06	0.2	-

S1.5.6. ρ, $\nabla^2\rho$ and ε at the BCP of the B-N bond for the last four steps of the final refinement strategy

Refinement step	Property	v_{total}	s_{total}	v_{mean}	s_{mean}	$\frac{\lvert v_{mean} - v_{total}\rvert}{s_{mean}}$	$\frac{s_{mean}}{s_{total}}$
17	ρ	0.747	0.011	0.749	0.005	0.4	0.4
18	ρ	0.745	0.011	0.747	0.004	0.5	0.4
19	ρ	0.748	0.011	0.750	0.005	0.3	0.5
20	ρ	0.779	0.011	0.781	0.006	0.4	0.5
17	$\nabla^2\rho$	1.86	0.05	1.8	0.4	0.2	7.9
18	$\nabla^2\rho$	1.95	0.05	1.9	0.4	0.2	7.9
19	$\nabla^2\rho$	1.76	0.05	1.7	0.4	0.2	8.2
20	$\nabla^2\rho$	0.08	0.05	0.1	0.4	0.1	8.5
17	ε	0.65	-	0.66	0.06	0.1	-
18	ε	0.73	-	0.73	0.06	0.0	-
19	ε	0.64	-	0.64	0.06	0.0	-
20	ε	0.58	-	0.61	0.06	0.4	-

S2. Evaluation of the method

S2.1. Cross validation of the final refinement strategy with 10 test sets

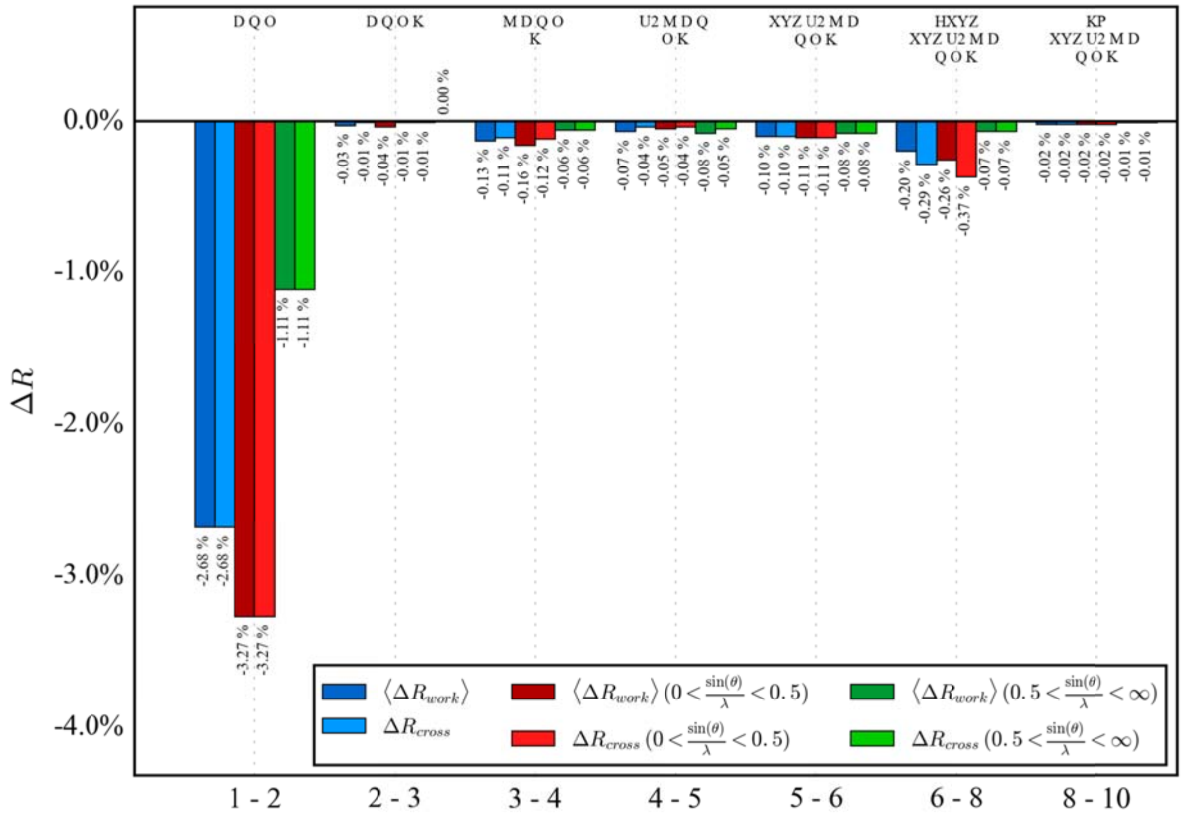

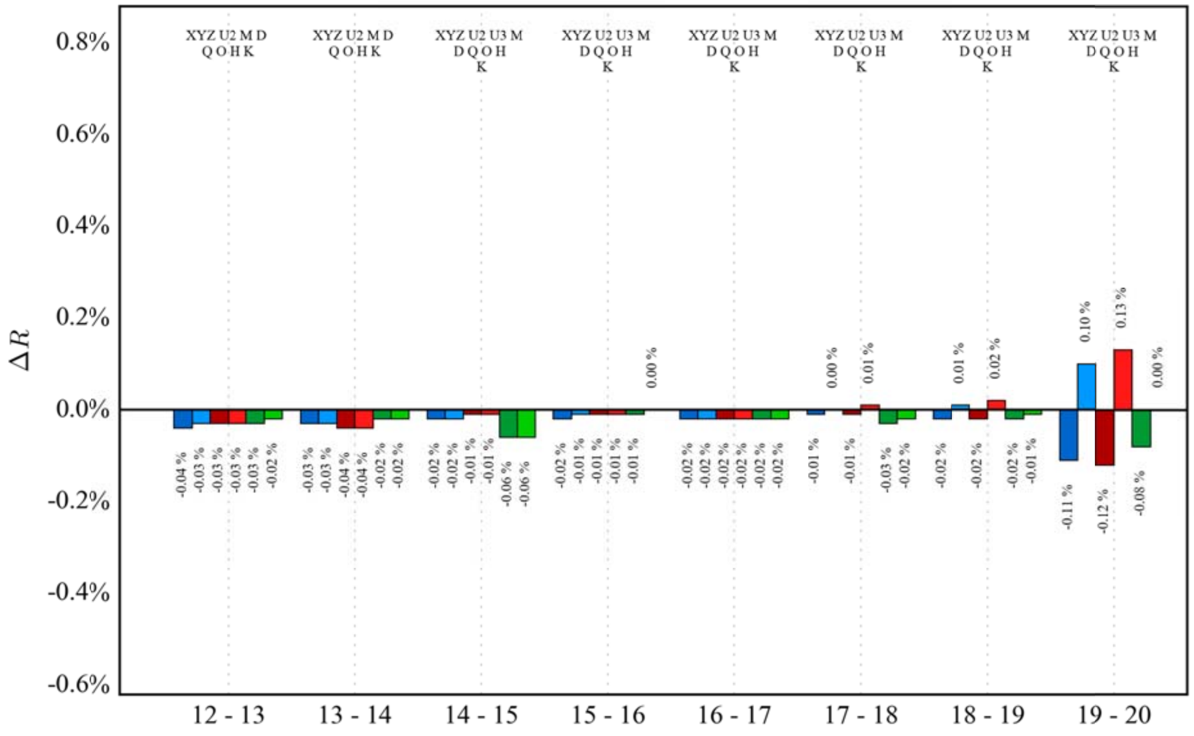

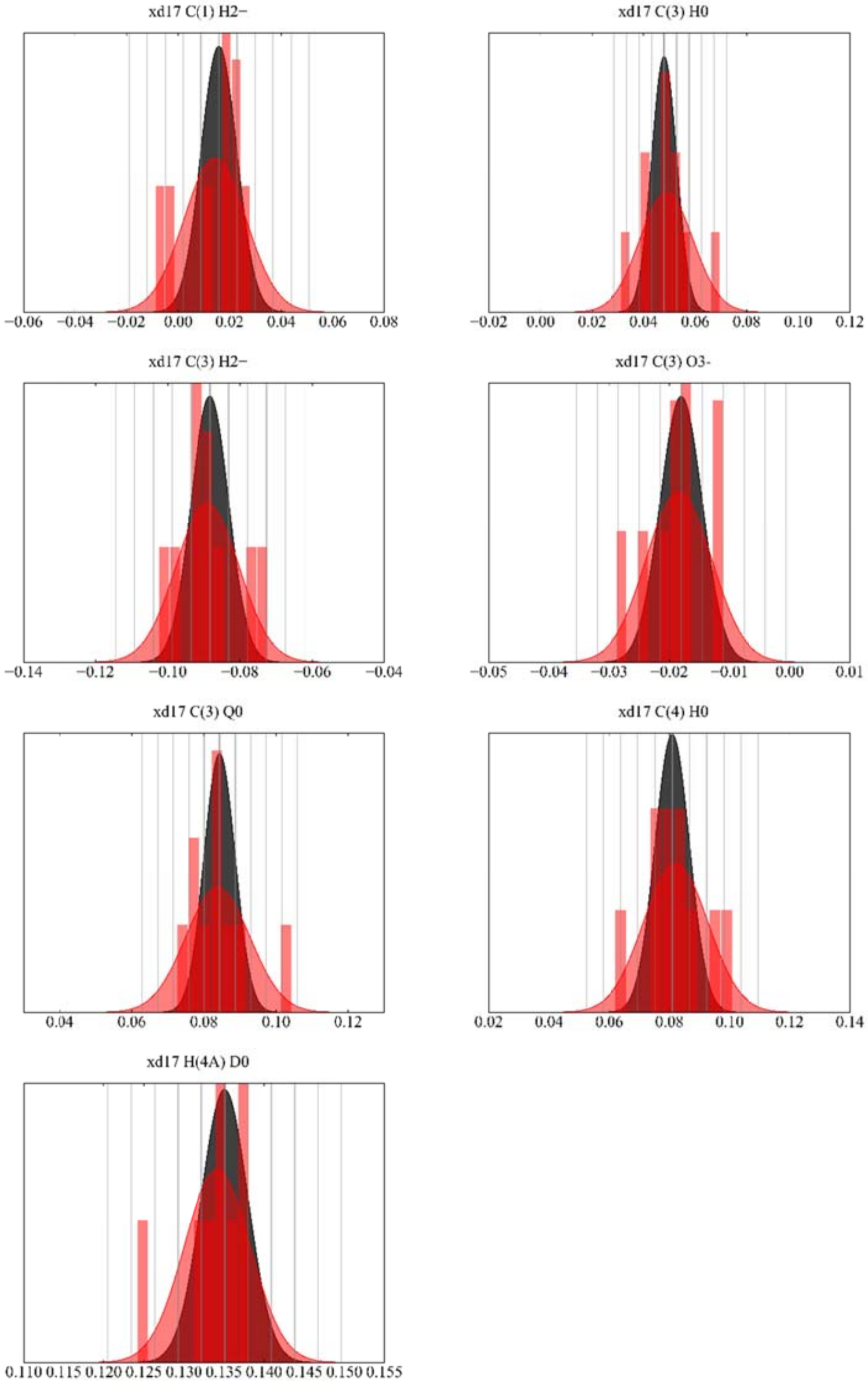

For 7 out of 618 parameters of the final refinement one to three v_i differ more than 3 σ from $v_{total.}$

S2.2. Cross validation of the final refinement strategy with 50 test sets

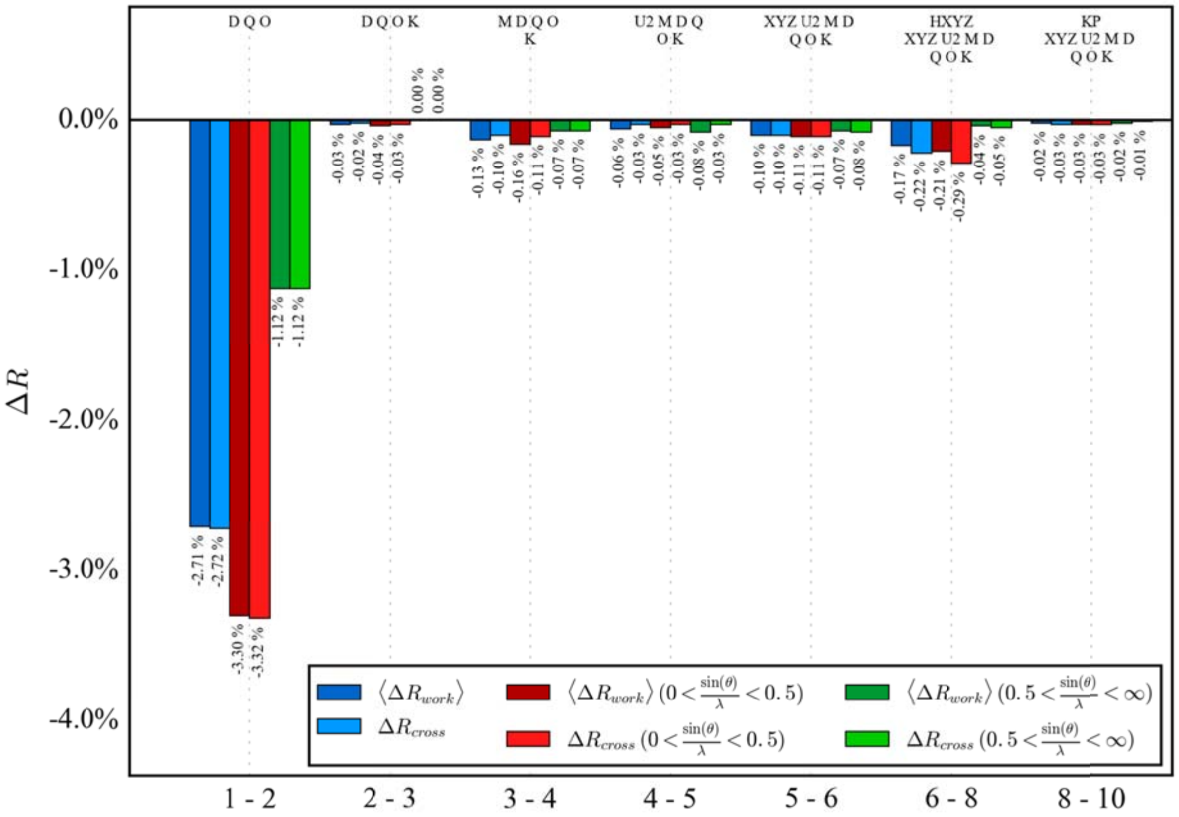

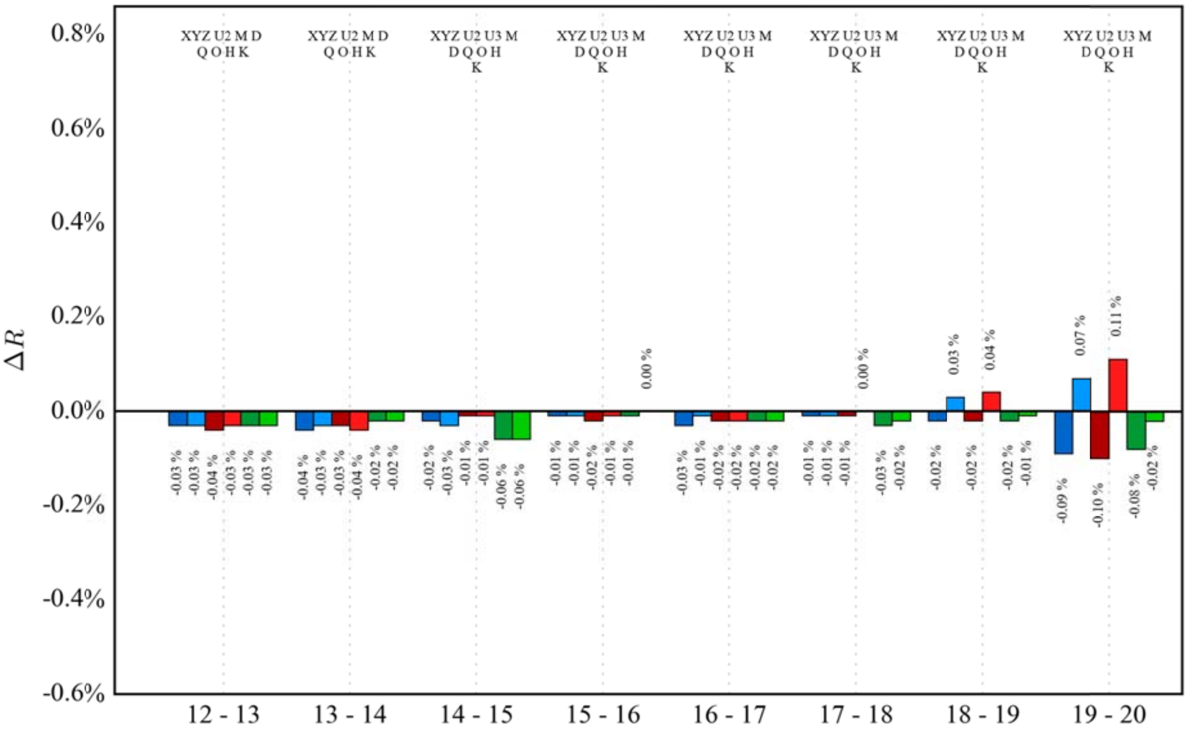

For none of 618 parameters of the final refinement a v_{i} differ more than 3 σ from $v_{\mathrm{total.}}$.

S2.3. Cross validation of the final refinement strategy with 20 test sets, but no special care on Friedel mates

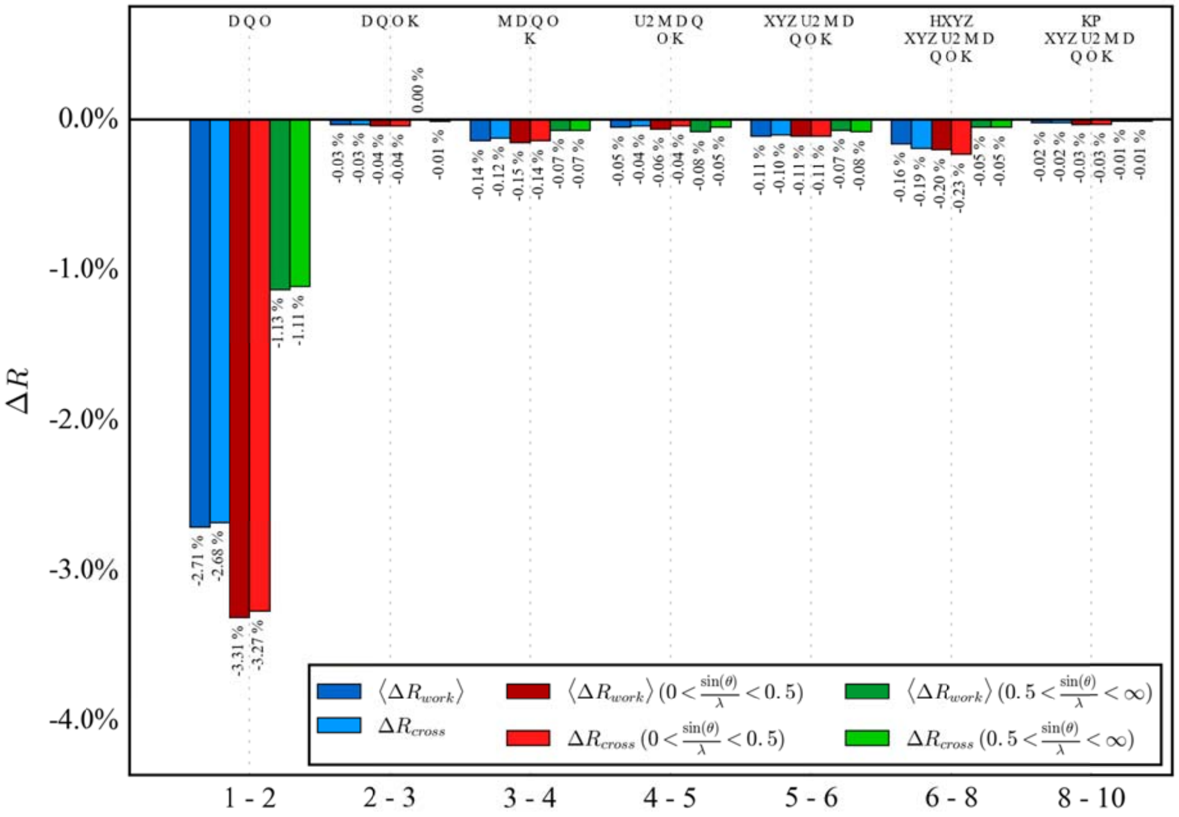

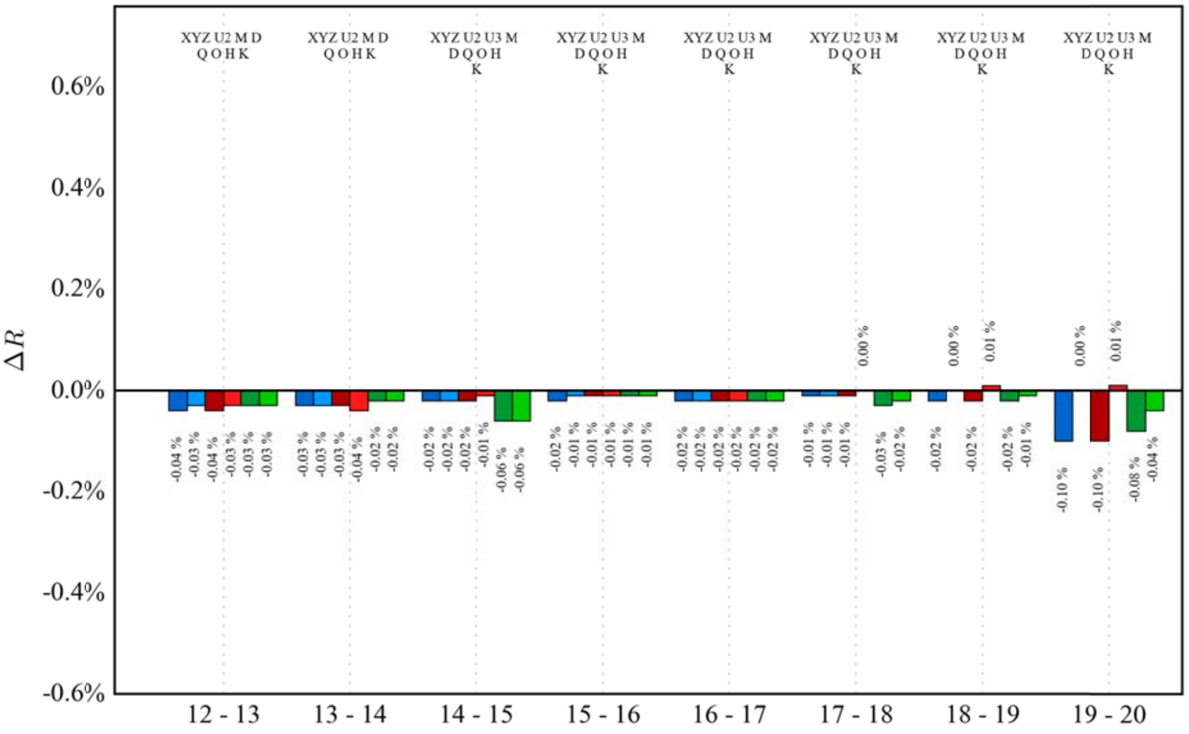

For 1 of 618 parameters of the final refinement one v_i differ more than 3 σ from v_{total}..

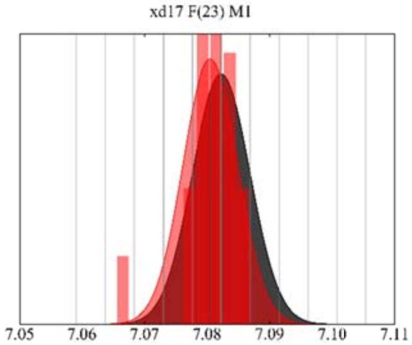

S3. Structure 2

S3.1. Local coordinate system

ATOM	ATOM0	AX1	ATOM1	ATOM2	AX2	Symm	CHEMCON	κ set
S(1)	P(1)	Z	S(1)	H(13)	Y	cyl		1
P(1)	C(1)	X	P(1)	S(1)	Y	mz		2
C(1)	P(1)	Z	C(1)	C(2)	Y	mm2		3
C(2)	C(5)	Z	C(2)	C(7)	Y	mm2		4
C(3)	C(6)	Z	C(3)	C(2)	Y	mm2		5
C(4)	C(7)	Z	C(4)	C(3)	Y	mm2		6
C(5)	C(2)	Z	C(5)	C(6)	Y	mm2	C(4)	6
C(6)	C(3)	Z	C(6)	C(7)	Y	mm2	C(3)	5
C(7)	C(4)	Z	C(7)	C(2)	Y	mm2	C(2)	4
C(8)	C(1)	Z	C(8)	C(7)	Y	mm2		7
C(9)	C(12)	Z	C(9)	C(14)	Y	mm2	C(2)	4
C(10)	C(13)	Z	C(10)	C(9)	Y	mm2	C(3)	5
C(11)	C(14)	Z	C(11)	C(10)	Y	mm2	C(4)	6
C(12)	C(9)	Z	C(12)	C(13)	Y	mm2	C(4)	6
C(13)	C(10)	Z	C(13)	C(14)	Y	mm2	C(3)	5
C(14)	C(11)	Z	C(14)	C(9)	Y	mm2	C(2)	4
C(15)	P(1)	X	C(15)	H(15)	Y	mz		8
C(16)	C(15)	Z	C(16)	H(16A)	Y	3m		9
C(17)	C(15)	Z	C(17)	H(17B)	Y	3m	C(16)	9
C(18)	P(1)	X	C(18)	H(18)	Y	mz	C(15)	8
C(19)	C(18)	Z	C(19)	H(19C)	Y	3m	C(16)	9
C(20)	C(18)	Z	C(20)	H(20C)	Y	3m	C(16)	9
H(3)	C(3)	Z	H(3)	C(4)	Y	cyl		10
H(4)	C(4)	Z	H(4)	C(3)	Y	cyl	H(3)	10
H(5)	C(5)	Z	H(5)	C(4)	Y	cyl	H(3)	10
H(6)	C(6)	Z	H(6)	C(5)	Y	cyl	H(3)	10
H(8)	C(8)	Z	H(8)	C(7)	Y	cyl	H(3)	10
H(10)	C(10)	Z	H(10)	C(11)	Y	cyl	H(3)	10
H(11)	C(11)	Z	H(11)	C(10)	Y	cyl	H(3)	10
H(12)	C(12)	Z	H(12)	C(13)	Y	cyl	H(3)	10
H(13)	C(13)	Z	H(13)	C(12)	Y	cyl	H(3)	10
H(15)	C(15)	Z	H(15)	H(3)	Y	cyl		11
H(16A)	C(16)	Z	H(16A)	H(16B)	Y	cyl		12
H(16B)	C(16)	Z	H(16B)	H(16A)	Y	cyl	H(16A)	12

H(16C)	C(16)	Z	H(16C)	H(16B)	Y	cyl	H(16A)	12
H(17A)	C(17)	Z	H(17A)	H(17C)	Y	cyl	H(16A)	12
H(17B)	C(17)	Z	H(17B)	H(17C)	Y	cyl	H(16A)	12
H(17C)	C(17)	Z	H(17C)	H(17A)	Y	cyl	H(16A)	12
H(18)	C(18)	Z	H(18)	C(19)	Y	cyl	H(15)	11
H(19A)	C(19)	Z	H(19A)	H(19C)	Y	cyl	H(16A)	12
H(19B)	C(19)	Z	H(19B)	H(19A)	Y	cyl	H(16A)	12
H(19C)	C(19)	Z	H(19C)	H(19A)	Y	cyl	H(16A)	12
H(20A)	C(20)	Z	H(20A)	H(20C)	Y	cyl	H(16A)	12
H(20B)	C(20)	Z	H(20B)	H(20C)	Y	cyl	H(16A)	12
H(20C)	C(20)	Z	H(20C)	H(20A)	Y	cyl	H(16A)	12

S3.2. Refinement strategy

Abbreviations: M: monopoles; D: dipoles; Q: quadrupoles; O: octupoles; H: hexadecapoles, U: Uij, k: kappa, nosym: no local symmetry constraints, nocon: no chemical constraint.

The scale factor is refined in every step but only mentioned in the first.

Step	Parameter	# data	*d*/*p*	d_l/p_m	$R(F^2)$
1	Scale factor	1	18435.0	-	5.49
2	DOQ H(only S, P)	60	307.3	29.5	2.69
3	MDOQ H(only S, P)	71	259.6	24.5	2.46
4	MDOQH(only S, P) U	203	90.8	24.5	1.76
5	MDOQH(only S, P)Uxyz	269	68.5	24.5	1.77
6	xyz(H)	70	24.9	-	1.31
7	MDOQH(only S, P) Uxyz[xyz(H)]	269	68.5	24.5	1.69
8	MDOQH(only S, P) Uxyzk	278	66.3	21.8	1.66
9	k’	10	1834.5	-	1.61
10	MDOQH(only S, P) Uxyzk[k’]	278	66.3	21.8	1.54
11	MDOQH(also C) Uxyzk	300	61.5	17.1	1.51
12	MDOQHUxyzk (anthracene: mm2→mx)	332	55.5	13.0	1.49
13	MDOQHUxyzk nosym	438	42.1	7.3	1.47
14	MDOQHUxyzk nosym nocon	763	24.2	3.1	1.41

d/*p*: data to parameter ratio, d_l/p_m: low-resolution ($\sin(\theta)/\lambda$) < 0.5 Å^{-1}) data to mono-, multipole ($\kappa^{(\prime)}$) parameter ratio

S3.3. Outlier

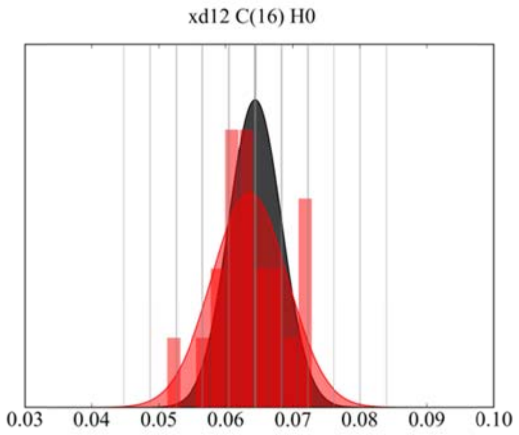

S4. Structure 3

S4.1. Local coordinate system

ATOM	ATOM0	AX1	ATOM1	ATOM2	AX2	R/L	Symm	CHEMCON	κ set
Cl(1)	P(1)	Z	Cl(1)	N(2)	Y	R	_cy		1
Cl(2)	P(1)	Z	Cl(2)	N(2)	Y	L	_cy	Cl(1)	1
Cl(3)	P(2)	Z	Cl(3)	N(1)	Y	L	_cy		2
Cl(4)	P(2)	Z	Cl(4)	N(1)	Y	R	_cy	Cl(3)	2
P(1)	N(2)	Z	P(1)	Cl(1)	Y	R	_mm2		3
P(2)	N(1)	Z	P(2)	Cl(3)	Y	L	_mm2		4
N(1)	P(2)	Z	N(1)	CL(3)	Y	L	_mm2		5
N(2)	P(1)	Z	N(2)	CL(1)	Y	R	_mm2		6
Cl(1)	P(1)	Z	Cl(1)	N(2)	Y	R	_mm2		6

Pole populations of P(1) and P(2), and N(1) and N(2), respectively, are constrained to be identical.

S4.2. Refinement strategy

Abbreviations: M: monopoles; D: dipoles; Q: quadrupoles; O: octupoles; H: hexadecapoles, U: Uij, k: kappa, , C(atom name): Gram Charlier 3rd order, nosym: no local symmetry constraints, nocon: no chemical constraint.

The scale factor is refined in every step but only mentioned in the first.

Step	Parameter	# param.	# data	d/p	d_l/p_m	σ cut off		$R(F^2)$
1	Scale factor	1	6406	6406	None	3		2.18
2	DOQH	21	6406	305.0	14.7	3		1.68
3	MDOQH	23	6406	278.5	12.78	3		1.64
4	MDOQHU	63	6406	101.7	12.78	3		1.45
5	MDOQHUxyz	83	6406	77.2	12.78	3		1.31
6	MDOQHUxyzk	86	6406	74.5	12.0	3		1.19
7	k’	4	6406	1601.5	None	3		1.16
8	MDOQHUxyzk [k’]	86	6406	74.5	12.0	3		1.11
9	MDOQHUxyzk [sig obs = 0)	86	7082	82.3	12.18	0		1.12
10	MDOQHUxyzkC(Cl)	118	7082	60.0	12.18	0		0.93
11	MDOQHUxyzkC(Cl,P)	134	7082	52.9	12.18	0		0.92
12	MDOQHUxyzkC(Cl,P,N)	150	7082	47.2	12.18	0		0.90
13	MDOQHUxyzkC(Cl,P,N)(Cl m)	160	7082	44.3	8.65	0		0.90
14	MDOQHUxyzkC(Cl,P,N)(Cl m) (P, N m)	172	7082	41.2	6.42	0		0.89
15	MDOQHUxyzkC(Cl,P,N)(Cl m) (P, N m) nocon	247	7082	28.7	4.85	0		0.88
16	MDOQHUxyzkC(Cl,P,N)(Cl m) (P, N m) nocon nosym	287	7082	24.7	3.66	0		0.88

d/*p*: data to parameter ratio, d_l/p_m: low-resolution ($\sin(\theta)/\lambda$) < 0.5 Å^{-1}) data to mono-, multipole ($\kappa^{(')}$) parameter ratio

S4.3. Cross-validation

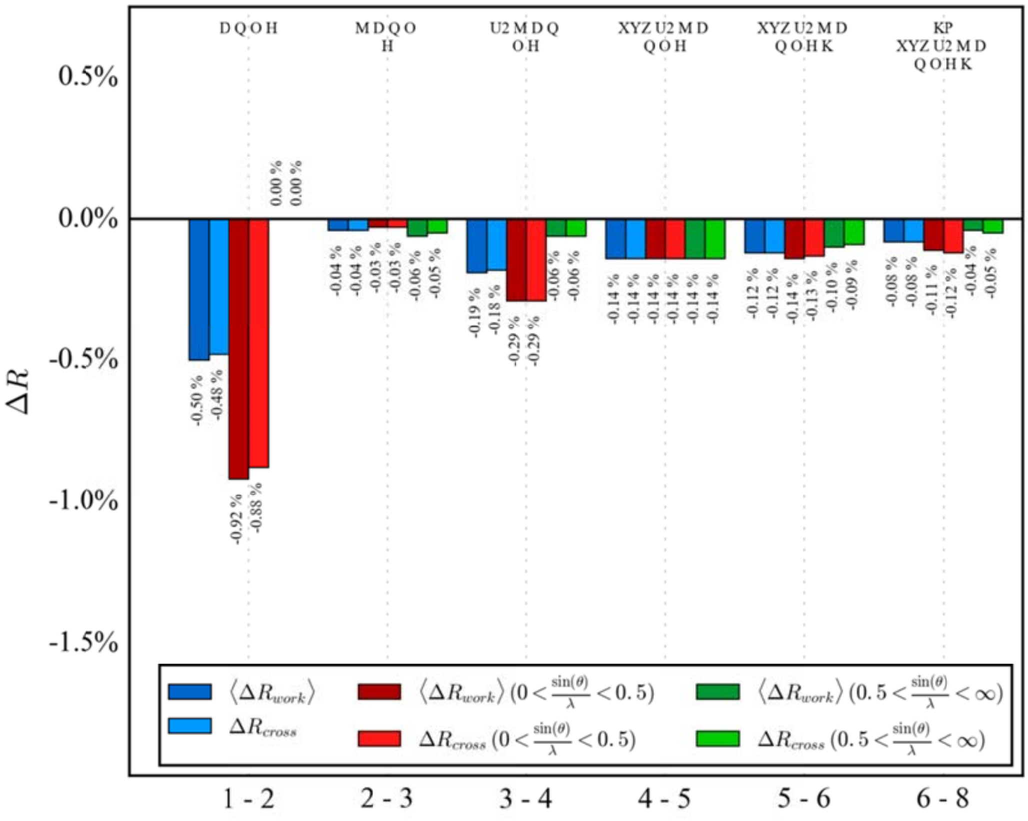

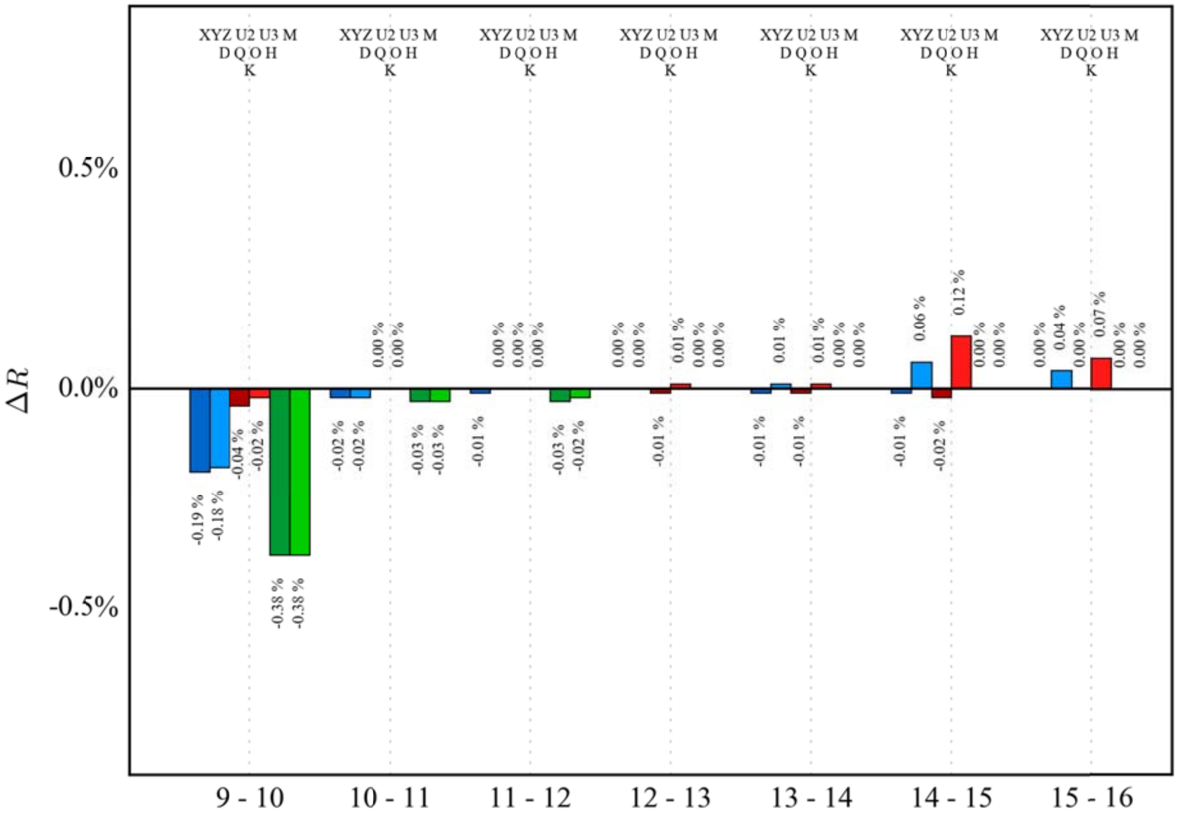

S5. Structure 4

S5.1. Refinement strategy

Abbreviations: M: monopoles; D: dipoles; Q: quadrupoles; O: octupoles; H: hexadecapoles, U: U_{ij}, k: kappa, C(atom name): Gram Charlier 3rd order, nosym: no local symmetry constraints, nocon: no chemical constraint.
The scale factor is refined in every step but only mentioned in the first.

Step	Parameter	# param.	# data	*d*/*p*	d_l/p_m	σ cut off	$R(F^2)$
1	Scale factor	1	24005	24005.0	None	3	6.21
2	DOQH	160	24005	150.0	16.88	3	3.47
3	DOQHk	167	24005	143.7	16.21	3	3.35
4	MDOQk	188	24005	127.7	13.77	3	3.16
5	MDOQkU	458	24005	52.4	13.77	3	3.07
6	MDOQUxyzk	593	24005	40.5	13.77	3	2.78
7	xyz(H)	55	2835	51.6	None	3	2.76
8	MDOQUxyzk [xyz(H)]	593	24005	40.5	13.77	3	2.76
9	k'	8	24005	3000.6	None	3	2.74
10	MDOQUxyzk [k']	593	24005	40.5	13.77	3	2.69
11	MDOQUxyzk [sigobs=0]	593	31139	52.5	14.68	0	2.79
12	MDOQHUxyzk C(F, C34-C36, C121, C141, C221, C241)	763	31139	40.8	14.68	0	2.50
13	MDOQHUxyzk C(FC C12, C13, C32, C33)	783	31139	39.8	14.68	0	2.47
14	MDOQHUxyzk C(some F, C), Ph only m (except para)	807	31139	38.6	13.15	0	2.46
15	MDOQHUxyzk C(some F, C) Ph only m (except para) P,N,C1,C2 nosym	857	31139	36.3	10.80	0	2.44
16	MDOQHUxyzk C(some F, C), Ph only m (except para) P,N,C1,C2 nosym, Fm	887	31139	35.1	9.76	0	2.44
17	MDOQHUxyzk C(some F, C), nosym	1037	31139	30.0	6.58	0	2.40
18	MDOQHUxyzk(FC anharm), nosym, nocon	1737	31139	17.9	2.61	0	2.20

d/*p*: data to parameter ratio, d_l/p_m: low-resolution ($\sin(\theta)/\lambda$) < 0.5 Å^{-1}) data to mono-, multipole ($\kappa^{(')}$) parameter ratio

S5.2. Cross validation

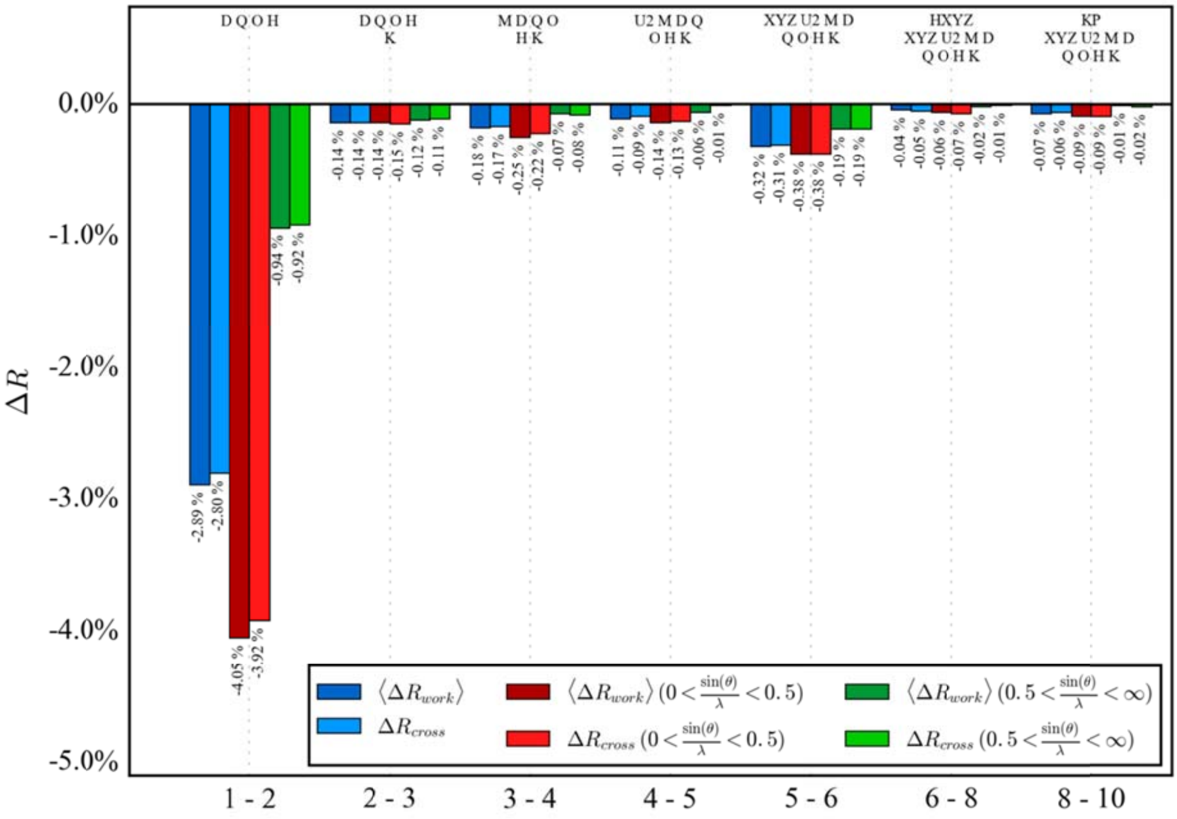

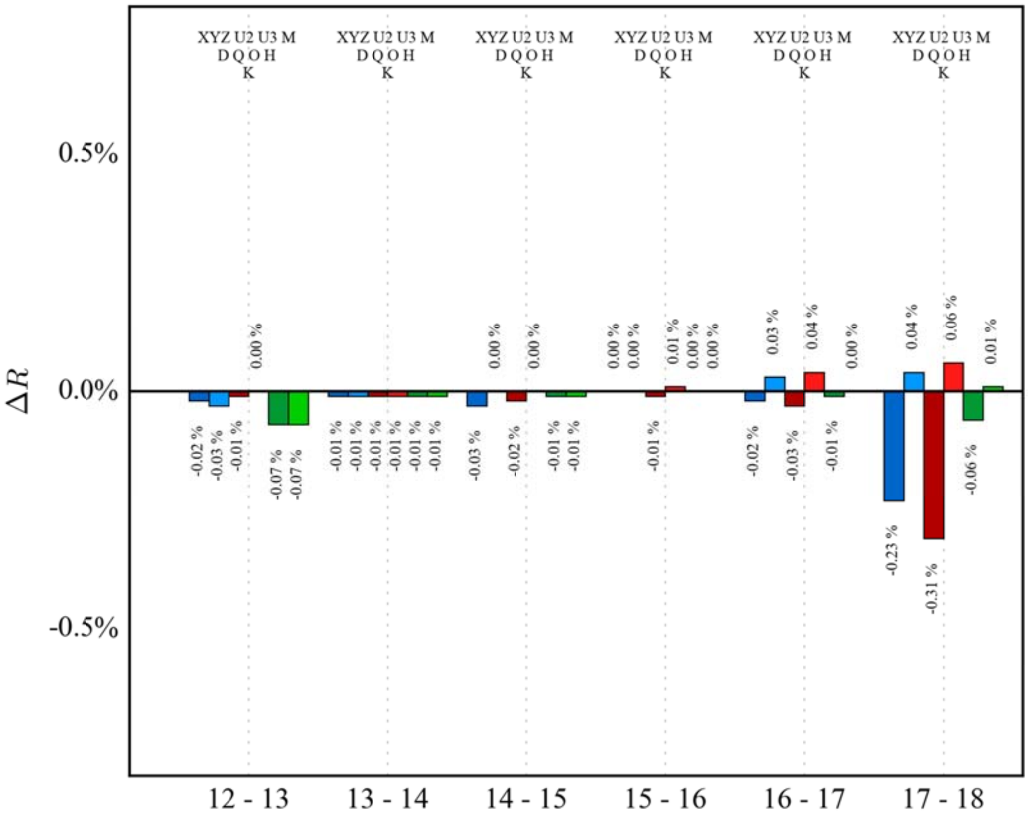

S5.3. DRK-Plots

S5.3.1. all data

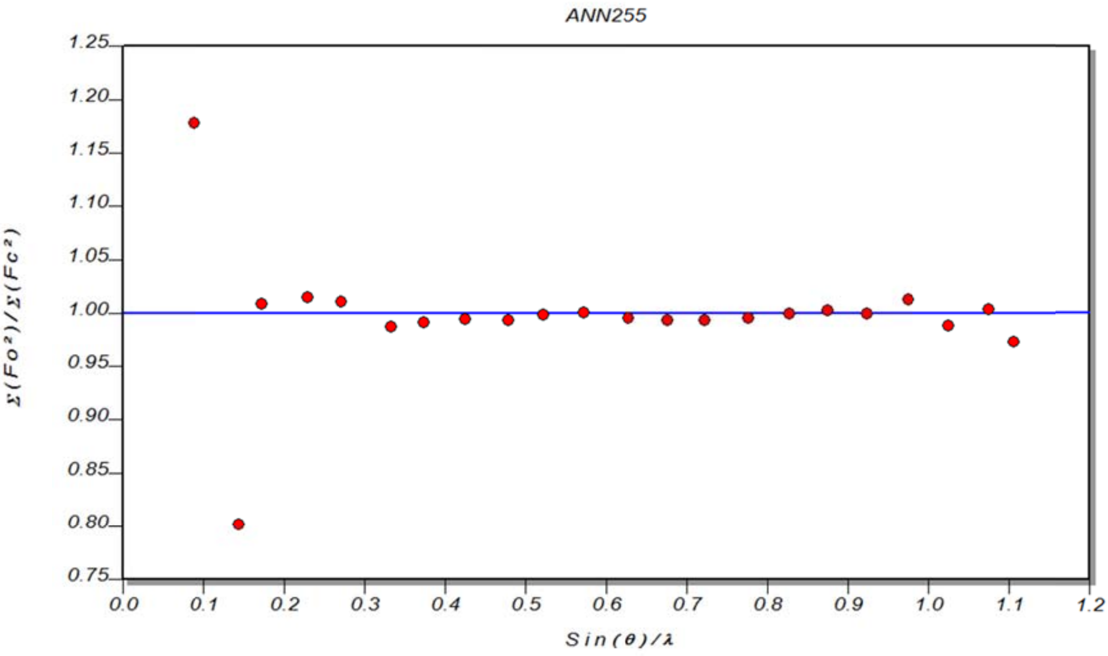

S5.3.2. after omission of two outlier reflections

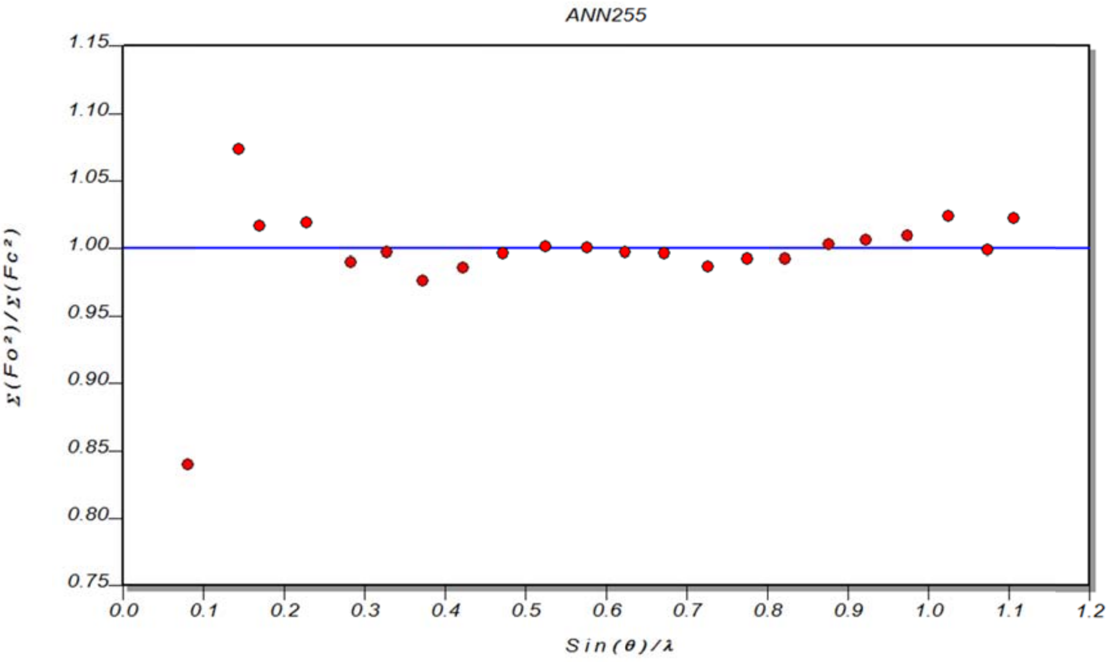

S6. Structure 5

S6.1. Local coordinate system

ATOM	ATOM0	AX1	ATOM1	ATOM2	AX2	Symm	CHEMCON	κ set
Si(1)	DUM1	X	Si(1)	C(1)	Y	R	mXmY2Z	
N(1)	C(1)	X	N(1)	C(4)	Y	R	mZ	
N(2)	C(24)	X	N(2)	C(27)	Y	R	mZ	N(1)
C(1)	C(2)	X	C(1)	N(1)	Y	R	NO	
C(2)	DUM14	Z	C(2)	C(1)	Y	R	mXmY2Z	
C(3)	DUM13	Z	C(3)	C(2)	Y	R	mXmY2Z	
C(4)	N(1)	X	C(4)	C(3)	Y	R	mZ	
C(5)	N(1)	Z	C(5)	C(6)	Y	R	mXmY2Z	
C(6)	C(5)	X	C(6)	C(7)	Y	L	mZ	
C(7)	C(6)	X	C(7)	C(8)	Y	L	mZ	
C(8)	C(5)	Z	C(8)	C(9)	Y	R	mXmY2Z	
C(9)	C(10)	X	C(9)	C(8)	Y	R	mZ	C(7)
C(10)	C(5)	X	C(10)	C(9)	Y	R	mZ	C(6)
C(11)	DUM2	Z	C(11)	C(12)	Y	R	mXmY2Z	
C(12)	DUM3	Z	C(12)	C(13)	Y	R	mXmY2Z	
C(13)	DUM4	Z	C(13)	C(14)	Y	R	mXmY2Z	
C(14)	DUM5	Z	C(14)	C(13)	Y	L	mXmY2Z	C(12)
C(15)	DUM6	Z	C(15)	C(14)	Y	L	mXmY2Z	C(11)
C(16)	C(4)	Z	C(16)	H(16B)	Y	L	3ZmX	
C(17)	C(4)	Z	C(17)	H(17B)	Y	R	3ZmX	C(16)
C(18)	DUM16	X	C(18)	C(6)	Y	L	mZ	
C(19)	C(18)	Z	C(19)	H(19A)	Y	L	3ZmX	
C(20)	C(18)	Z	C(20)	H(20A)	Y	R	3ZmX	C(19)
C(21)	DUM17	X	C(21)	C(10)	Y	R	mZ	C(18)
C(22)	C(21)	Z	C(22)	H(22C)	Y	R	3ZmX	C(19)
C(23)	C(21)	Z	C(23)	H(23C)	Y	L	3ZmX	C(19)
C(24)	C(25)	X	C(24)	N(2)	Y	R	NO	C(1)
C(25)	DUM15	Z	C(25)	C(24)	Y	R	mXmY2Z	C(2)
C(26)	DUM18	Z	C(26)	C(25)	Y	R	mXmY2Z	C(3)
C(27)	N(2)	X	C(27)	C(26)	Y	R	mZ	C(4)
C(28)	N(2)	Z	C(28)	C(29)	Y	R	mXmY2Z	C(5)
C(29)	C(28)	X	C(29)	C(30)	Y	L	mZ	C(6)
C(30)	C(29)	X	C(30)	C(31)	Y	L	mZ	C(7)
C(31)	C(28)	Z	C(31)	C(32)	Y	R	mXmY2Z	C(8)
C(32)	C(33)	X	C(32)	C(31)	Y	R	mZ	C(7)
C(33)	C(28)	X	C(33)	C(32)	Y	R	mZ	C(6)
C(34)	DUM7	Z	C(34)	C(35)	Y	R	mXmY2Z	C(11)
C(35)	DUM8	Z	C(35)	C(36)	Y	R	mXmY2Z	C(12)
C(36)	DUM9	Z	C(36)	C(37)	Y	R	mXmY2Z	C(13)

C(37)	DUM10	Z	C(37)	C(36)	Y	L	mXmY2Z	C(12)
C(38)	DUM11	Z	C(38)	C(37)	Y	L	mXmY2Z	C(11)
C(39)	C(27)	Z	C(39)	H(39A)	Y	L	3ZmX	C(16)
C(40)	C(27)	Z	C(40)	H(40B)	Y	R	3ZmX	C(16)
C(41)	DUM19	X	C(41)	C(29)	Y	L	mZ	C(18)
C(42)	C(41)	Z	C(42)	H(42A)	Y	L	3ZmX	C(19)
C(43)	C(41)	Z	C(43)	H(43A)	Y	R	3ZmX	C(19)
C(44)	DUM21	X	C(44)	C(33)	Y	R	mZ	C(18)
C(45)	C(44)	Z	C(45)	H(45A)	Y	R	3ZmX	C(19)
C(46)	C(44)	Z	C(46)	H(46B)	Y	L	3ZmX	C(19)
C(47)	C(48)	Z	C(47)	H(47C)	Y	R	3ZmX	
C(48)	DUM20	Z	C(48)	C(49)	Y	R	mXmY2Z	
C(49)	DUM12	Z	C(49)	C(47)	Y	R	mXmY2Z	

S6.2. Refinement strategy

Abbreviations: M: monopoles; D: dipoles; Q: quadrupoles; O: octupoles; H: hexadecapoles, U: U_{ij}, k: kappa, C(atom name): Gram Charlier 3rd order, nosym: no local symmetry constraints, nocon: no chemical constraint.

The scale factor is refined in every step but only mentioned in the first.

Step	Parameter	# param.	# data	*d*/*p*	d_l/p_m	σ cut off	$R(F^2)$
1	Scale factor	1	38908	38908	None	3	5.51
2	M	20	38908	1945.4	209.29	3	5.38
3	MDOQH	214	38908	181.8	20.44	3	3.32
4	MDOQHU	526	38908	74.0	20.44	3	3.18
5	MDOQHUxyz	682	38908	57.0	20.44	3	2.80
6	MDOQHUxyzk	691	38908	56.3	18.86	3	2.63
7	xyz(H)	232	4392	18.9	None	3	2.05
8	MDOQHUxyzk [xyz(H)]	691	38908	56.3	18.86	3	2.43
9	k’	10	38908	3890.8	None	3	2.39
10	MDOQHUxyzk [k’]	691	38908	56.0	18.86	3	2.35
11	MDOQHUxyzk [sig obs = 0)	691	46112	66.7	19.41	0	2.41
12	MDOQHUxyzkC	831	46112	55.5	19.41	0	2.23
13	MDOQHUxyzkC(Si mm2→ mx)	837	46112	55.1	18.92	0	2.22
14	MDOQHUxyzk (Si nosym)	847	46112	54.4	18.16	0	2.22
15	MDOQHUxyzk (mm2→ m)	901	46112	51.2	14.93	0	2.20
16	MDOQHUxyzk (nocon carbene)	986	46112	46.8	11.66	0	2.19
17	MDOQHUxyzk nosym	1215	46112	38.0	7.33	0	2.14
18	MDOQHUxyzk nosym nocon	1920	46112	24.0	3.42	0	2.05

d/*p*: data to parameter ratio, d_l/p_m: low-resolution ($\sin(\theta)/\lambda$) < 0.5 Å^{-1}) data to mono-, multipole ($\kappa^{(')}$) parameter ratio

S6.3. Cross validation

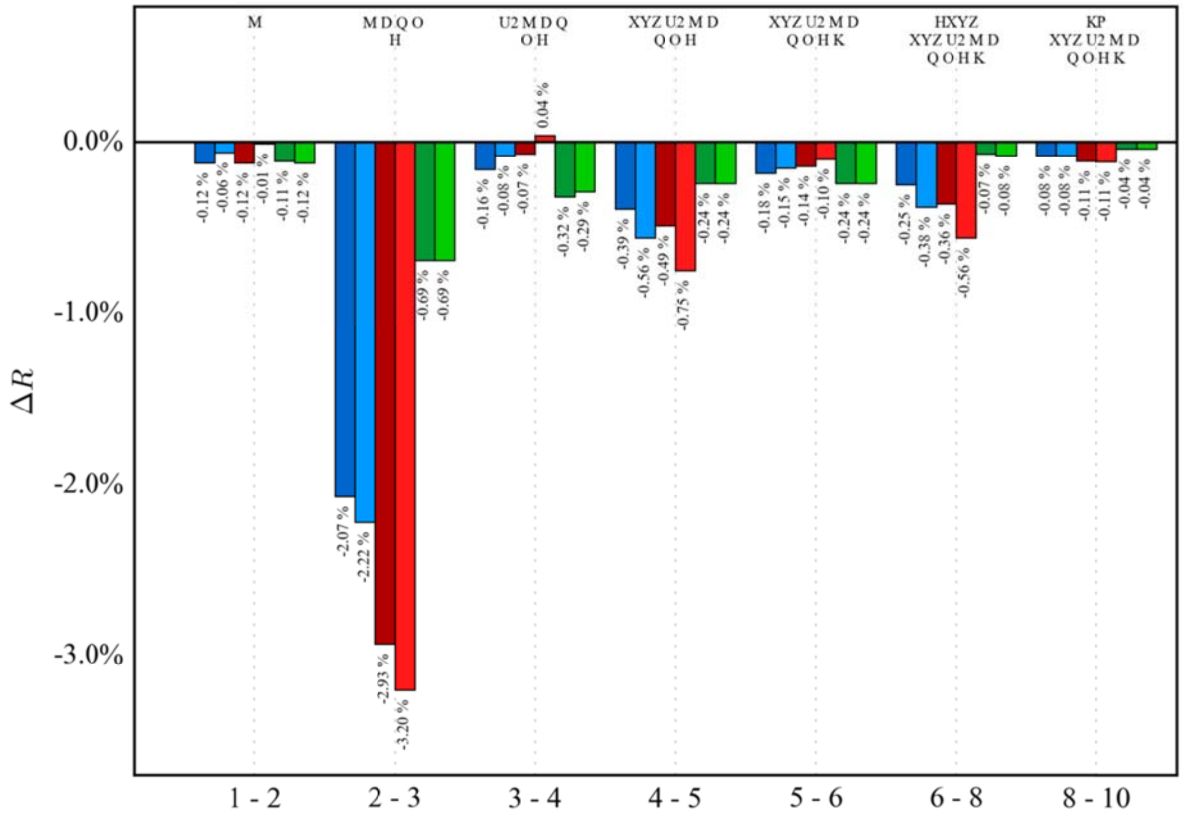

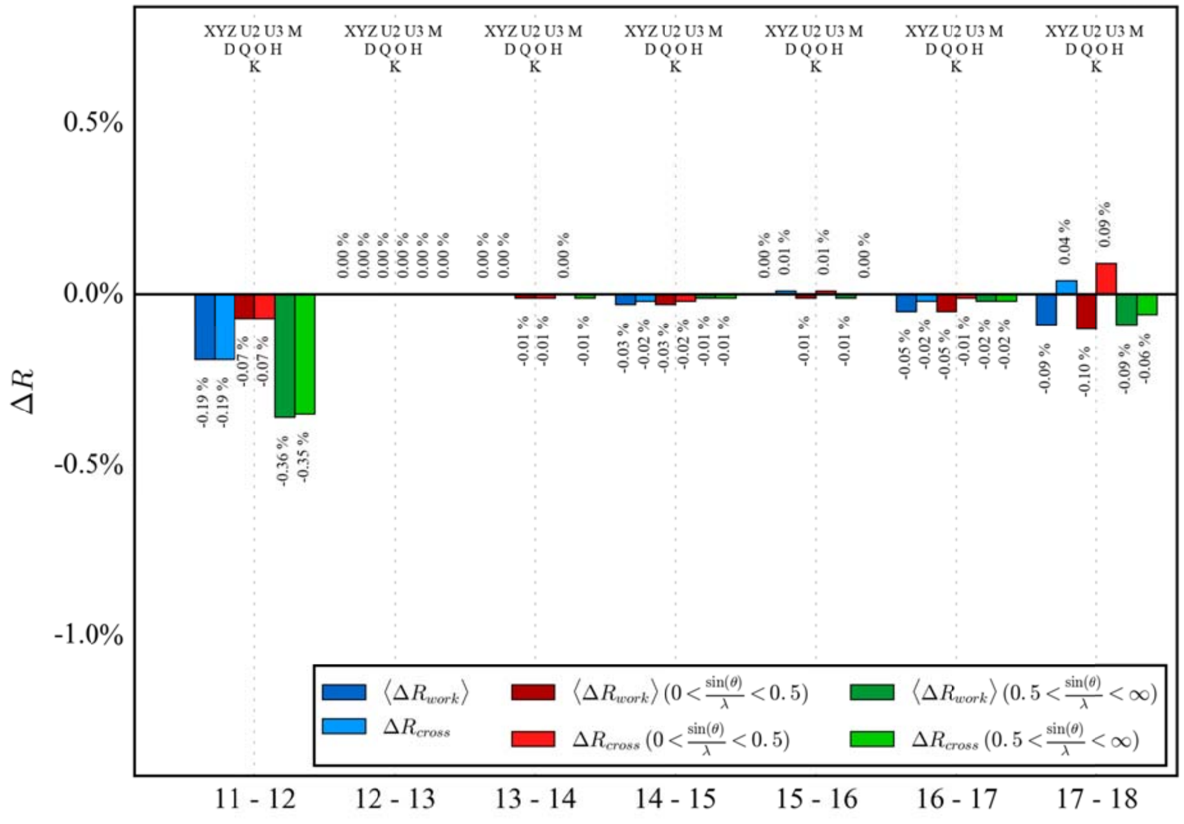

Supplement: Supplementary file 4 [file m-04-00420-sup4.pdf]
